# Yeast Pol4 Promotes Tel1-Regulated Chromosomal Translocations

**DOI:** 10.1371/journal.pgen.1003656

**Published:** 2013-07-18

**Authors:** Jose F. Ruiz, Benjamin Pardo, Guillermo Sastre-Moreno, Andrés Aguilera, Luis Blanco

**Affiliations:** 1 Centro de Biología Molecular “Severo Ochoa”, Universidad Autónoma de Madrid/CSIC, Madrid, Spain; 2 Centro Andaluz de Biología Molecular y Medicina Regenerativa CABIMER, Universidad de Sevilla, Sevilla, Spain; Duke University, United States of America

## Abstract

DNA double-strand breaks (DSBs) are one of the most dangerous DNA lesions, since their erroneous repair by nonhomologous end-joining (NHEJ) can generate harmful chromosomal rearrangements. PolX DNA polymerases are well suited to extend DSB ends that cannot be directly ligated due to their particular ability to bind to and insert nucleotides at the imperfect template-primer structures formed during NHEJ. Herein, we have devised genetic assays in yeast to induce simultaneous DSBs in different chromosomes *in vivo*. The repair of these breaks *in trans* could result in reciprocal chromosomal translocations that were dependent on classical Ku-dependent NHEJ. End-joining events leading to translocations were mainly based on the formation of short base pairing between 3′-overhanging DNA ends coupled to gap-filling DNA synthesis. A major proportion of these events were specifically dependent on yeast DNA polymerase Pol4 activity. In addition, we have discovered that Pol4-Thr^540^ amino acid residue can be phosphorylated by Tel1/ATM kinase, which could modulate Pol4 activity during NHEJ. Our data suggest that the role of Tel1 in preventing break-induced chromosomal translocations can, to some extent, be due to its stimulating effect on gap-filling activity of Pol4 to repair DSBs in *cis*. Overall, this work provides further insight to the molecular mechanisms of DSB repair by NHEJ and presents a new perspective to the understanding of how chromosomal translocations are formed in eukaryotic cells.

## Author Summary

Chromosomal translocations are one of the most common types of genomic rearrangements, which may have a relevant impact on cell development. They are often generated from DNA double-strand breaks that are inaccurately repaired by DNA repair machinery. In this study, we have developed genetic assays in yeast to analyze the molecular mechanisms by which these translocations can arise. We found evidence showing that the classical nonhomologous end-joining repair pathway can be a source of chromosomal translocations, with a relevant role for yeast DNA polymerase Pol4 in such processes. The involvement of Pol4 is based on its efficient gap-filling DNA synthesis activity during the joining of overhanging DNA ends with short sequence complementarity. In addition, we discovered that DNA polymerase Pol4 can be modified during the repair of the breaks via phosphorylation by Tel1 kinase. This phosphorylation seems to have important structural and functional implications in the action of Pol4, which can finally influence the formation of translocations. This work provides a useful tool for deciphering factors and mechanisms involved in DNA double-strand break repair and identifying the molecular pathways leading to chromosomal translocations in eukaryotic cells.

## Introduction

DNA double-strand breaks (DSBs) are one of the most cytotoxic lesions. They can originate during cellular metabolism or upon exposure to DNA damaging agents such as radiation or chemicals. DSBs can be repaired by two main mechanisms, homologous recombination (HR) or nonhomologous end-joining (NHEJ) [1]. In the absence of DNA homology, NHEJ is the main source of chromosomal translocations in both yeast [2] and mammalian cells [3], [4]. In the latter, those translocations generated as byproducts of V(D)J and class switch recombination in B cells are particularly relevant, since they can promote cancer, especially leukemia and lymphoma [5], [6]. Despite the ability of NHEJ to join breaks directly, most DSBs occurring *in vivo* are not fully complementary or have chemical modifications at their ends, and cannot be directly ligated. In these cases, additional processing, such as DNA end trimming or gap-filling DNA synthesis, may be required in order to optimize base pairing before ligaton [7]. The extent of DSB end processing influences the speed of repair and defines the existence of two forms of NHEJ. Classical NHEJ (c-NHEJ) is the fastest and most conservative form, as it relies on a limited degradation of DNA ends. On the other hand, the alternative NHEJ pathway (alt-NHEJ) relies on an extensive end resection that exposes hidden sequence microhomologies surrounding DNA ends to be rejoined. Core components of c-NHEJ are the Ku70/80 and XRCC4/DNA Ligase IV complexes (YKu70/80 and Lif1/Dnl4 in yeast, respectively) [7], [8]. In vertebrates, Ku is part of a larger complex called DNA-dependent protein kinase (DNA-PK), whose catalytic subunit is DNA-PKcs kinase. The Ku complex initially mediates the synapsis between the two broken DNA ends, protecting them from extensive degradation. Thereafter, it also recruits other components, such as the XRCC4/DNA Ligase IV complex. In the absence of Ku, or due to its departure from DSB ends, the occurrence of alt-NHEJ increases relative to the extent of DSB resection, as it allows uncovering larger microhomologies to be used for end-joining [9].

NHEJ also involves accessory factors such as DNA polymerases belonging to the PolX family [10]. Among mammalian PolX polymerases, Polλ and Polμ are specialized DNA polymerases with a large capacity to use imperfect template-primer DNA substrates. Thus, they are able to extend DNA ends that cannot be directly ligated by NHEJ, as demonstrated *in vitro* with human whole-cell extracts [11]. This is mainly due to their capability of simultaneously binding both the 5′ and 3′ ends of small DNA gaps, which permits an efficient gap-filling [12], [13]. Based on such DNA binding properties, these polymerases can efficiently search for sequence microhomologies and utilize DNA substrates with unpaired bases at or near the 3′-terminus [14]–[16]. These scenarios are frequent in NHEJ when DNA ends have extremely low sequence complementarity. PolX polymerases are specifically recruited to DSBs during NHEJ by interacting with Ku and XRCC4/DNA Ligase IV through their BRCT domains [17], [18]. This interaction allows gap-filling during end-joining reactions, as demonstrated both *in vitro* [18]–[20] and *in vivo* [21]–[24]. Whereas mammalian cells have four PolX polymerases (Polλ, Polμ Polβ, and TdT), in yeast there is a unique member, Pol4. Yeast Pol4 combines most of the structural and biochemical features of its mammalian counterparts Polλ and Polμ [25], [26], including the BRCT-mediated interaction with core NHEJ factors [27]. It has been shown that Pol4 is required to recircularize linear plasmids having terminal microhomology, as an example of NHEJ reactions performed *in vivo* [28]–[31]. In addition, Pol4 is involved in NHEJ-mediated repair of chromosomal DSBs induced in *cis* [32]–[34], and in NHEJ reactions where no base complementarity between DSB ends is available [29].

Here we have devised intron-based assays in yeast to generate two simultaneous DSBs in different chromosomes *in vivo*, whose repair by NHEJ could generate reciprocal chromosomal translocations. End joining events leading to translocations were mainly based on the formation of short base pairing between 3′-overhanging ends coupled to gap-filling. A major proportion of these events were specifically dependent on yeast DNA polymerase Pol4, as the DNA synthesis-mediated repair signature disappeared in *pol4Δ* cells. Other results, suggesting that Tel1-mediated suppression of translocations can be in part due to Pol4 regulation to promote DNA synthesis-dependent NHEJ, will be also discussed.

## Results

### A genetic system to analyze NHEJ-mediated chromosomal translocations in yeast

**Figure 1:**
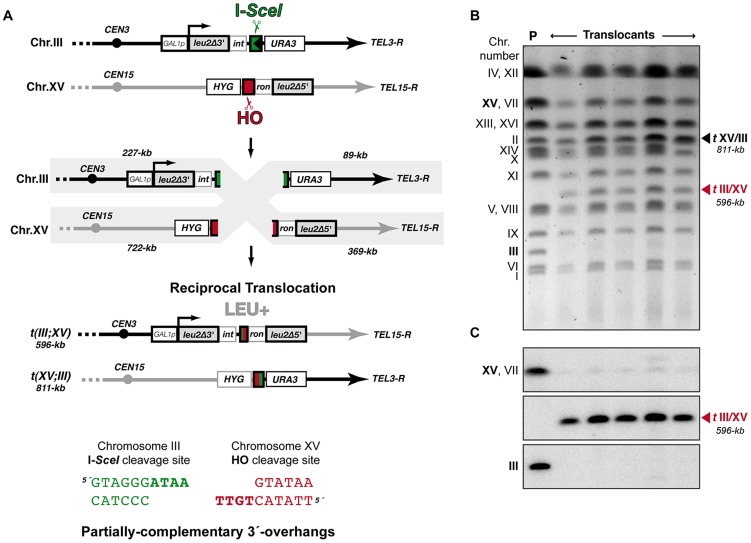
Intron-based assay to detect NHEJ-mediated chromosomal translocations in yeast. (A) Scheme of the assay. Two nonhomologous halves of *LEU2* gene (*leu2Δ5′* and *leu2Δ3′*) were integrated at chromosomes XV and III, respectively. Downstream of the *leu2Δ3′* fragment, which is under control of the *GAL1* promoter, it was inserted one copy of the I-*SceI* cut site. The *leu2Δ5′* fragment is preceded by the HO endonuclease cut site. Induced DSBs at chromosomes III and XV can be repaired generating a reciprocal chromosomal translocation that restores a functional *LEU2* gene with a functional *ACT1* intron inside. The length of chromosomal fragments after cleavage and the size of new translocated chromosomes generated are indicated. *(Bottom)* Cleavage by HO and I-*SceI* endonucleases generates 4-nt long 3′-overhanging DNA ends. (B, C) Molecular karyotype of wild-type Leu+ translocants analyzed by pulsed-field gel electrophoresis (PFGE). (B) Ethidium bromide staining of gels. The electrophoretic mobility of natural yeast chromosomes is indicated. Parental strain (P) is shown as a reference. After DSBs induction, two new translocated chromosomes of 596-kb (*t*III/XV, marked with a red triangle) and 811-kb (*t*XV/III, marked with a black triangle) were detected. Parental chromosomes III and XV (marked in bold on the left) simultaneously disappeared. Chromosomes XV and VII have the same electrophoretic mobility in our experimental conditions. (C) Southern analysis. PFGE gels were analyzed by Southern using a *LEU2* specific probe. After DSB induction, *LEU2* signal was specifically detected in the smaller translocated chromosome (*t*III/XV, marked with a red triangle). Concomitantly, *LEU2* signal disappeared in parental chromosomes III and XV.

**Figure 2:**
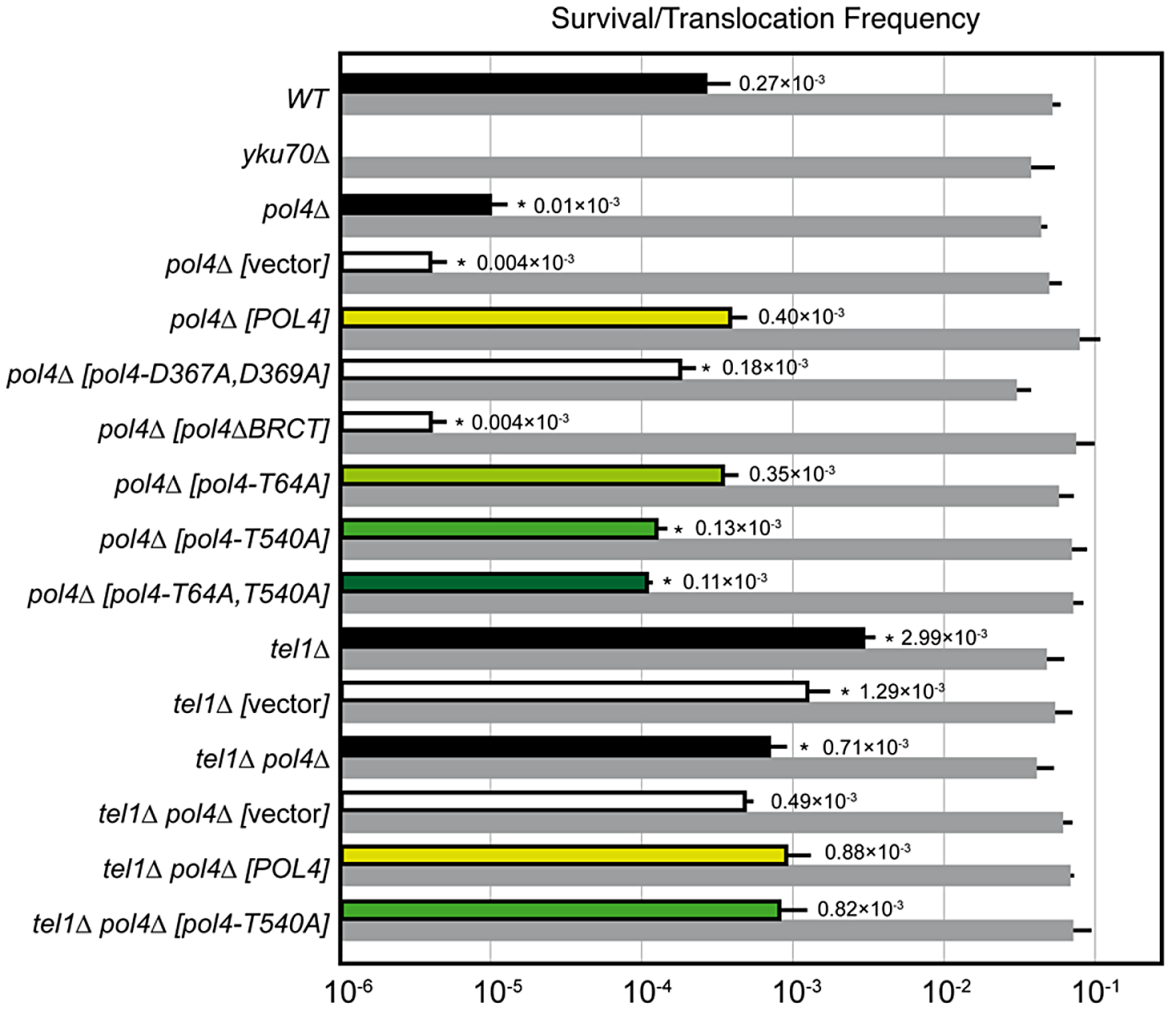
NHEJ-mediated repair of DSBs with partially-complementary overhangs. Wild-type and indicated mutant yeast strains were subjected to two simultaneous DSBs by continuous expression of both I-*SceI* and HO by switching growth conditions from glucose- to galactose-containing media. Total cell survival (Gal/Glu, grey bars) and Leu+ translocant frequency among total cells (Gal Leu+/Glu, black, white and colored bars) are plotted on a logarithmic scale. Data represent the median plus standard deviation from at least four independent experiments. Statistically significant lower or higher values with respect to either wild-type (WT) or *pol4Δ* [*POL4*] complemented strains are marked with an asterisk (*p<0.001 by the Mann-Whitney test).

We have modified a previously reported yeast genetic assay [35] to analyze the repair mechanism through which two induced DSBs can be joined by NHEJ to form chromosomal translocations. The system is mainly based on two nonhomologous halves of the *LEU2* gene (*leu2*Δ*5′* and *leu2Δ3′*), each one fused to either an HO or I-*SceI* endonuclease cleavage site and integrated into a different chromosome (Figure 1A). In the experimental conditions used, DSBs were induced by continuous expression of both endonucleases in cells accumulated in the G1 phase of the cell cycle, when NHEJ is the predominant DSB repair pathway. NHEJ-mediated repair of DSBs can generate reciprocal translocations that restore a functional *LEU2* gene and can be selected as Leu+ colonies in selective plates. Within the *LEU2* gene, translocation breakpoints are embedded in a functional intronic sequence that can tolerate the variability produced during NHEJ (Figure 1A). Breakpoints can be further analyzed by PCR amplification and DNA sequencing, and the repair events can then be deduced. After DSB induction, Leu+ translocants were obtained at a frequency of 0.27×10^−3^ in a wild-type strain (Figure 2 and Table S1). The electrophoretic karyotyping of wild-type Leu+ translocants, as determined by pulsed-field gel electrophoresis (PFGE), verified the expected molecular nature of translocations. Thus, ethidium bromide staining of gels and Southern analysis with both *LEU2* and *HYG* specific probes showed two new 596- and 811-kb long chromosomes resulting from reciprocal translocations (Figure 1 and Figure S1). *LEU2* signal was specifically detected in the smaller translocated chromosome, which carried the joined *LEU2* halves (Figure 1C). Simultaneously, an *HYG* signal was specifically detected in the larger translocated chromosome (Figure S1). No Leu+ translocants were recovered in the absence of Yku70 (Figure 2), demonstrating that translocations were mediated by c-NHEJ. These results validated our assay to analyze the genetic requirements and mechanisms leading to chromosomal translocations via c-NHEJ.

### Breakpoint sequence analysis indicates a preferential use of short base pairing at DNA ends coupled to gap-filling

**Figure 3:**
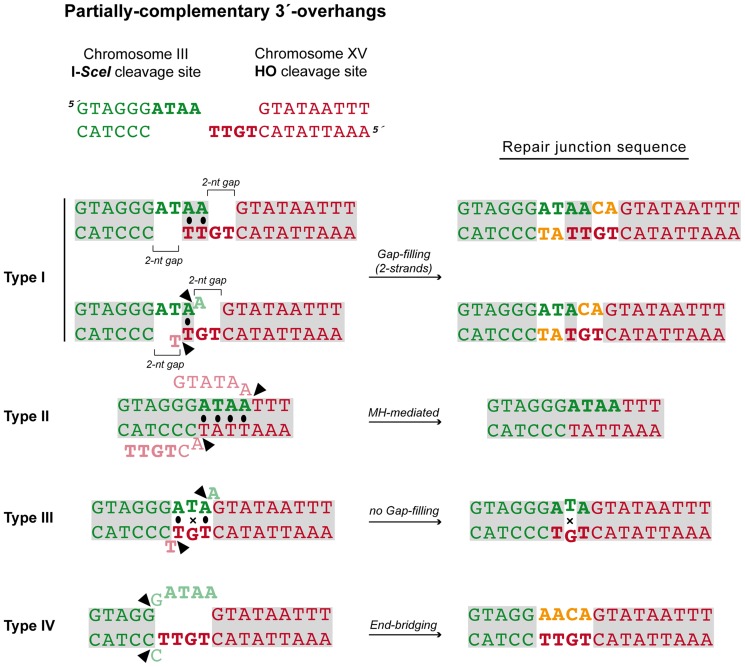
NHEJ repair types of DSBs with partially-complementary ends. All strands are depicted with the canonical 5′-to-3′ orientation. The 4-nucleotide 3′-protruding single-stranded DNA ends generated after both I-*SceI* (green) and HO (red) cleavage are shown in bold and the base pairing that can be established is marked with black dots. Mismatches are indicated with an X. Complementary sequences are shown in grey boxes. Inserted nucleotides are shown in orange. Action of nucleases is depicted as black triangles. Resected nucleotides are represented as semitransparent letters.

After the induction of endonucleases cleavage, 4-nt long 3′-protruding DSB ends with partial complementarity were generated (Figure 1A). To unravel the molecular events leading to NHEJ-mediated translocations, we analyzed the breakpoints of 24 independent wild-type Leu+ translocants by sequencing *ACT1* intron within the reconstituted *LEU2* gene (all sequencing data are available in Figure S2). This analysis showed a major proportion of repair events based on the formation of either 1-nt or 2-nt base pairing between the 3′-protruding DSB ends, which generated 2-nt gaps on both strands (Type I, 67% of the events; Figure 3 and Table 1). These small gaps should necessarily be filled-in through a templated insertion (+CA/+AT), as occurs in NHEJ-mediated repair of DSBs induced *in cis* [36]. The second more represented repair event in wild-type cells (Type II, 21%) involved the use of short (4-nt) microhomologies between one 3′-protruding DNA end and adjacent sequences in the other DSB end for base pairing (Figure 3). A third type of repair in wild-type cells (Type III, 8%) implied the formation of a 3-nt base pairing between the two 3′-protruding DSB ends and the exonucleolytic removal of the terminal nucleotides (Figure 3). These DSBs could then be directly ligated without the need of gap-filling. Type III events would involve the formation of a T:G mismatch, which should be processed later by mismatch repair machinery (Figure 3). Finally, a less frequent repair type (Type IV, 4%) implied the degradation of one 3′-protruding end to generate a blunt end. This could be utilized as a primer in a DNA synthesis reaction that used the other intact 3′-protruding end as a template in an end-bridging*-like* reaction (Figure 3) [37]. These results indicated a major role for gap-filling-mediated repair of induced DSBs leading to translocations in our experimental system.

**Table 1:** Repair types of DSBs with partially-complementary overhangs.

Strain	Leu+ Sequenced	Type I	Type II	Type III	Type IV
		*Gap-filling-mediated*	*MH-mediated*	*Direct ligation with no gap-filling*	*End-bridging*
WT	24	67% (16)	21% (5)	8% (2)	4% (1)
*yku70Δ*	0	nd	nd	nd	nd
*pol4Δ*	21	0	57% (12)	38% (8)	5% (1)
*pol4Δ* [*POL4*]	35	68% (24)	6% (2)	26% (9)	0
*pol4Δ* [*pol4*-*D367A,D369A*]	17	0	18% (3)	70% (12)	12% (2)
*pol4Δ* [*pol4ΔBRCT*]	18	0	39% (7)	50% (9)	11% (2)
*pol4Δ* [*pol4*-*T64A*]	35	74% (26)	0	14% (5)	11% (4)
*pol4Δ* [*pol4*-*T540A*]	36	36%*** (13)	17% (6)	28% (10)	19% (7)
*pol4Δ* [*pol4*-*T64A,T540A*]	0	nd	nd	nd	nd
*tel1Δ*	27	33%*** (9)	22% (6)	15% (4)	30% (8)
*tel1Δ pol4Δ*	23	26% (6)	17% (4)	43% (10)	13% (3)
*tel1Δ pol4Δ* [*POL4*]	30	43% (13)	23% (7)	23% (7)	10% (3)
*tel1Δ pol4Δ* [*pol4*-*T540A*]	30	27% (8)	20% (6)	37% (11)	16% (5)

Asterisks indicate statistically significant differences using a chi-squared test (***p<0.005; *tel1Δ* compared to WT; *pol4Δ* [*pol4*-*T540A*] compared to *pol4Δ* [*POL4*]); nd, no data).

### Yeast Pol4 promotes NHEJ-dependent chromosomal translocations

DNA polymerase Pol4, the only member of PolX family in yeast, synthesizes DNA efficiently from 3′-protruding ends that are annealed to form small gaps during classical NHEJ. As shown in Figure 2, we observed a significant decrease in the frequency of translocations in our assays when Pol4 was absent (0.27 *vs.* 0.01, 27-fold decrease, p<0.001). This suggested a relevant role for Pol4 in NHEJ-mediated repair leading to translocations. In agreement, *pol4*Δ cells completely lost gap-filling-mediated repair events (Type I; Table 1). Intriguingly, these cells did not lose type IV events, which also implied DNA synthesis for repair. Ectopic overexpression of *POL4* gene restored wild-type translocation frequency (Figure 2 and Table S1). Importantly, cells overexpressing wild-type Pol4 repaired induced DSBs mainly by gap-filling-mediated repair, as wild-type cells did (Table 1). This result validated the use of this overexpression system for the analysis of Pol4 mutants *in vivo*. Translocation frequency was partially dependent of Pol4 DNA polymerase activity, as it was reduced (0.40 *vs.* 0.18, 2-fold decrease, p<0.001) when we overexpressed a catalytically inactive Pol4 mutated at two of the three aspartic residues required for polymerization (*pol4-D367A,D369A* mutant; Figure 2). This reduction was even higher (4-fold, p<0.001) under more physiological conditions in *pol4*Δ cells expressing a catalytically inactive Pol4 from the *POL4* endogenous promoter (Figure S3). Notably, *pol4*Δ [*pol4-D367A,D369A*] cells did not show gap-filling-mediated repair events (Type I), thus confirming the role of the Pol4 polymerization activity during translocations formation (Table 1). It has been shown that a functional BRCT domain is strictly required for the recruitment of Pol4 to DSBs *in vivo* to catalyze gap-filling during NHEJ [27], [28], [32]. Accordingly, the overexpression of a Pol4*ΔBRCT* mutant protein in *pol4*Δ cells strongly inactivated Pol4 function during NHEJ-mediated repair of induced DSBs in our assays. These cells showed a similar translocation frequency level to *pol4*Δ cells and no gap-filling-mediated repair events (Type I; Figure 2 and Table 1). It is worth noting that the overexpression of *POL4* alleles in *pol4*Δ cells induced a strong increase of direct ligation repair events, which did not imply gap-filling (Type III, see Table 1). Altogether, these results suggested that Pol4 played a major role in the joining of DSBs with partial complementarity by filling the small DNA gaps present on both strands during NHEJ.

### Pol4 is phosphorylated by Tel1

**Figure 4:**
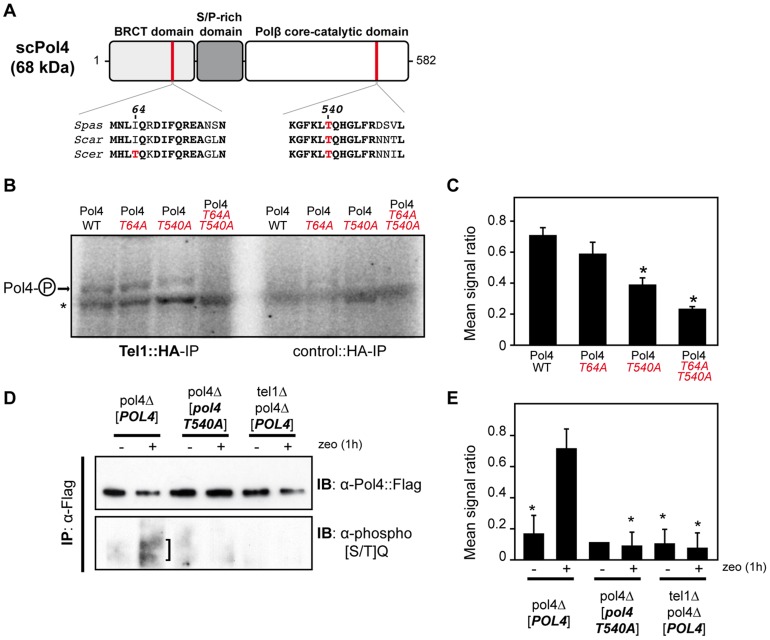
Pol4 phosphorylation by Tel1 kinase. (A) Pol4 structural and functional domains. The location of the two Pol4 [S/T]Q consensus motifs for Tel1 kinase activity is indicated. Amino acid alignment of these motifs in three different *Saccharomyces* species is shown below. Thr^64^ and Thr^540^ amino acid residues are marked in red. *Spas*, *Saccharomyces pastorianus*; *Scar*, *Saccharomyces cariocanus*; *Scer*, *Saccharomyces cerevisiae*. (B) *In vitro* kinase assay. Partially purified Pol4 proteins were subjected to kinase assays using HA-immunoprecipitates obtained from yeast cells either transformed (Tel1::HA-IP, *left*) or non-transformed (control::HA-IP, *right*) with a *TEL1::HA*- encoding plasmid. Phosphorylated Pol4 proteins are indicated with an arrow. A contaminant protein, showing basal levels of phosphorylation in all samples, is marked with an asterisk. (C) Quantitative measurement of Pol4 phosphorylation *in vitro* by immunoprecipitated Tel1. Quantification data are represented as ratio averages between phosphorylated Pol4 and phosphorylation of the contaminant protein. Error bars represent standard deviations. Statistical analysis was carried out using unpaired *t*-test with Welch's correction, compared to wild-type Pol4 phosphorylation (p values expressed as *p<0.05 were considered significant). (D) Detection of Pol4 phosphorylation *in vivo*. Flag-tagged Pol4 proteins were immunoprecipitated from G1-synchronized cells in the absence (−) or presence (+) of zeocin (zeo) to induce DSBs. After immunoprecipitation with anti-Flag antibodies, Flag-tagged proteins were detected with either anti-Flag antibodies (*upper panel*) or specific antibodies recognizing phosphorylated [SQ/TQ] motifs (*bottom panel*). Damage-induced SQ/TQ phosphorylation corresponding to Pol4 is indicated with a vertical bar. IB, immunoblotting; IP, immunoprecipitation. (E) Quantitative measurement of Tel1-mediated Pol4 phosphorylation *in vivo*. Quantification data are represented as ratio averages between Pol4 phosphorylation signals from the anti-phospho [SQ/TQ] immunoblotting and Pol4 signals from the anti-Flag immunoblotting. Error bars represent standard deviations. Statistical analysis was carried out using unpaired *t*-test with Welch's correction compared to Pol4 phosphorylation obtained in *pol4Δ* [*POL4*] cells treated with zeocin (p values expressed as *p<0.05 were considered significant).

Yeast Tel1 (homolog of mammalian ATM) is a serine/threonine protein kinase that is recruited and activated by DSBs. It has been reported that the absence of Tel1/ATM increases break-induced chromosomal translocations, likely due to a defect in DSB end tethering and resection [38], [39]. This finding was confirmed in our experimental system, as the frequency of translocations in *tel1*Δ cells significantly increased over wild-type level (2.99 *vs.* 0.27, 11-fold increase, p<0.001; Figure 2). Interestingly, the analysis of repair types in *tel1*Δ translocants showed a different repair pattern compared to wild-type, which included a significant decrease in gap-filling-mediated repair reactions (Type I) (from 67% to 33%, p<0.005; Table 1). Concomitantly, end-bridging reactions and those reactions that did not involve gap-filling increased in *tel1Δ* cells (Table 1). Thus, we asked whether Pol4 could be a target of Tel1/ATM during NHEJ-mediated DSB repair. We searched for potential Tel1 phosphorylation sites in the amino acid sequence of Pol4, and we found two threonine residues (Thr^64^ and Thr^540^) within [S/T]Q consensus sites, which have been defined for all PIIK-kinases, including Tel1 (Figure 4A). The carboxy-terminal T^540^Q consensus motif is highly conserved in different *Saccharomyces* species, probably reflecting its functional relevance (Figure 4A). To know whether Tel1 phosphorylates any of these threonine residues we partially purified His-tagged wild-type and mutant Pol4 proteins where the Thr^64^ and Thr^540^ amino acids were mutated to non-phosphorylatable alanines (Figure S4A). We analyzed their phosphorylation *in vitro* using HA-Tel1-enriched immunoprecipitates obtained as previously described [40] (Figure 4B and Figure S4B). Control immunoprecipitates from cells that were not transformed with the HA-Tel1-encoding plasmid were also used to detect the possible activities of other kinases (Figure 4B). We observed that *in vitro* phosphorylation of Pol4 was clearly higher when using Tel1-enriched immunoprecipitates than with those obtained from non-transformed cells (Figure 4B). As deduced from quantification of phosphorylation signals, wild-type Pol4 and mutant Pol4-*T64A* proteins were similarly phosphorylated by Tel1 (Figure 4C). However, a significant decrease of Pol4 phosphorylation was observed in the Pol4-*T540A* mutant, which was even higher in the Pol4-*T64A,T540A* double mutant (Figure 4C). These results indicated that Pol4-Thr^540^ residue is the most efficiently phosphorylated by Tel1 *in vitro*.

Next, we sought to determine if Pol4 phosphorylation also occurred in response to DSBs *in vivo*. For this purpose, Flag-tagged wild-type and *T540A* Pol4 proteins were overexpressed in *pol4Δ* cells in which we simultaneously induced DSBs with zeocin (Figure 4D). To promote NHEJ processing, DSBs were induced in G1-arrested cells. Flag-tagged Pol4 proteins were immunoprecipitated with anti-Flag antibodies and subsequently immunodetected using both anti-Flag antibodies and antibodies that specifically recognize phosphorylated SQ/TQ motifs. As shown in Figure 4D, a damage-induced SQ/TQ phosphorylation signal was specifically observed in *pol4Δ* [*POL4*] cells, which was detected as a slower migrating protein with respect to Pol4 molecular mass. Importantly, such a phosphorylation was barely detected when the Pol4*-T540A* phosphomutant was overexpressed in the same experimental conditions (Figure 4D). To further verify that the observed phosphorylation signal was dependent on Tel1, wild-type Pol4 was overexpressed in a *tel1Δ pol4Δ* double mutant. As expected, damage-induced SQ/TQ phosphorylation was again much weaker than that obtained in *pol4Δ* [*POL4*] cells, confirming its dependence on Tel1 (Figure 4D). As deduced from the quantification of phosphorylation signals, the decrease of damage-induced Pol4 phosphorylation either in the *pol4Δ* [*pol4-T540A*] mutant or in the absence of Tel1 kinase was statistically significant (Figure 4E). Altogether, our data suggested that Pol4 can be phosphorylated on Thr^540^ residue by Tel1 in response to DSBs.

### Tel1-mediated Pol4 phosphorylation influences DNA synthesis-dependent NHEJ responsible for chromosomal translocations

To determine the relevance of Tel1-mediated Pol4 phosphorylation *in vivo*, we analyzed the effect of overexpressing the different non-phosphorylatable Pol4 proteins in our system. Both translocation frequency and repair events observed in *pol4Δ* [*pol4-T64A*] mutants were similar to those observed in *pol4Δ* [*POL4*] cells (Figure 2 and Table 1). Interestingly, *pol4Δ* [*pol4-T540A*] mutants showed a significant reduction in the frequency of translocations compared to control cells (0.40 *vs.* 0.13, 3-fold decrease, p<0.001; Figure 2 and Table S1). This reduction was even stronger (7-fold, p<0.001) under a more physiological situation by expressing the Pol4-*T540A* phosphomutant from the *POL4* endogenous promoter (Figure S3). Overexpression of a double phosphomutant (*pol4-T64A,T540A*) generated a translocation frequency similar to that obtained in *pol4Δ* [*pol4-T540A*] single mutant, confirming that Pol4-Thr^64^ residue is not involved in the regulation of Pol4 activity (Figure 2 and Table S1). The molecular analysis of repair events in *pol4Δ* [*pol4-T540A*] mutants showed that the repair of the induced DSBs mainly occurred as in *tel1Δ* cells (Table 1). Notably, this included a 2-fold decrease in gap-filling-mediated repair (Type I) events compared to control conditions (from 68% to 36%, p<0.005; Table 1). Simultaneously, an increase in microhomology-mediated repair (Type II) and end-bridging repair (Type IV) was observed (Table 1). To further investigate the genetic interaction between Tel1 and Pol4 phosphorylation in our assays, we analyzed *tel1Δ pol4Δ* double mutants. According to the involvement of Pol4 in the formation of translocations, we observed a significant decrease of translocation frequency in *tel1Δ pol4Δ* cells compared with *tel1Δ* single mutants (0.71 *vs.* 2.99, 4-fold decrease, p<0.001, Figure 2 and Table S1). This decrease was lower than that observed in *pol4Δ* cells compared to wild type (0.01 *vs.* 0.27, 27-fold decrease, p<0.001), consistent with the presence of a basal level of gap-filling-mediated repair in the t*el1Δ pol4Δ* double mutants (Table 1). The overexpression of wild-type Pol4 complemented the absence of Pol4 in *tel1Δ pol4Δ* cells, as deduced by comparing *tel1Δ pol4Δ* [*POL4*] cells and *tel1Δ* cells carrying an empty vector (0.88 *vs.* 1.29; Table S1). The analysis of repair types in *tel1Δ pol4Δ* [*POL4*] cells showed a significant decrease of type I events (from 68% to 43%; p<0.005; Table 1) and a concomitant increase in type IV events (from 0% to 10%; p<0.005; Table 1) when compared to *pol4Δ* [*POL4*], similar to what occurred in *tel1Δ* cells. Finally, both the translocation frequencies and the types of repair in *tel1Δ pol4Δ* [*POL4*] and *tel1Δ pol4Δ* [*pol4-T540A*] were similar (Figure 2 and Table 1), which demonstrated the epistatic relationship between *tel1Δ* and *pol4-T540A* mutations. Together, these results indicated that the phosphorylation of Pol4-Thr^540^ by Tel1 stimulated Pol4-mediated gap-filling synthesis during NHEJ repair of DSBs with partial complementarity.

### Pol4 phosphorylation also promotes DNA synthesis-mediated NHEJ when DSB ends are non-complementary

**Figure 5:**
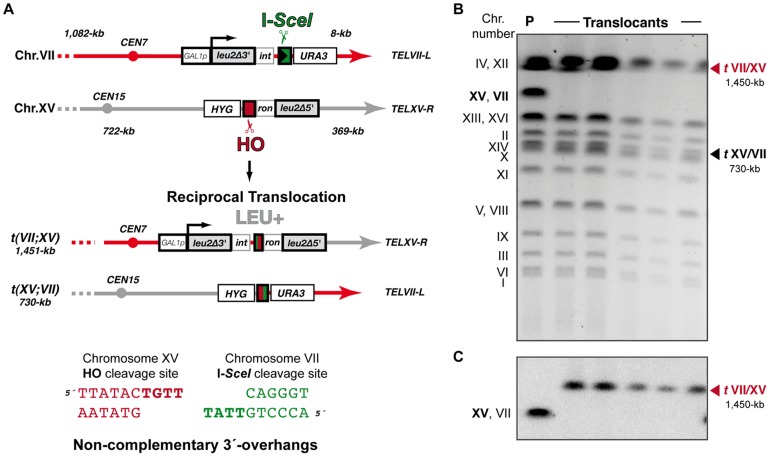
Intron-based assay to detect NHEJ-mediated chromosomal translocations in the absence of sequence complementarity. (A) Scheme of the assay. In this system the I-*SceI* endonuclease cleavage site was introduced at chromosome VII in an inverse orientation with respect to system represented in Figure 1. Cleavage by HO and I-*SceI* endonucleases generates 4-nt long 3′-overhanging ends completely non-complementary *(bottom)*. (B, C) Molecular karyotype of wild-type Leu+ translocants analyzed by PFGE (B) Ethidium bromide staining of gels. The electrophoretic mobility of natural yeast chromosomes is indicated. Parental strain (P) is shown as a reference. After DSBs induction, two new translocated chromosomes of 1,450-kb (*t*VII/XV, marked with a red triangle) and 730-kb (*t*XV/VII, marked with a black triangle) were detected. Parental chromosomes VII and XV (bold), which have the same electrophoretic mobility in the experimental conditions used here, simultaneously disappeared. (C) Southern analysis. PFGE gels were analyzed by Southern using a *LEU2* specific probe. After DSB induction, *LEU2* signal was specifically detected in the largest translocated chromosome (*t*VII/XV, marked with a red triangle). Concomitantly, *LEU2* signal disappeared in parental chromosomes VII and XV.

**Figure 6:**
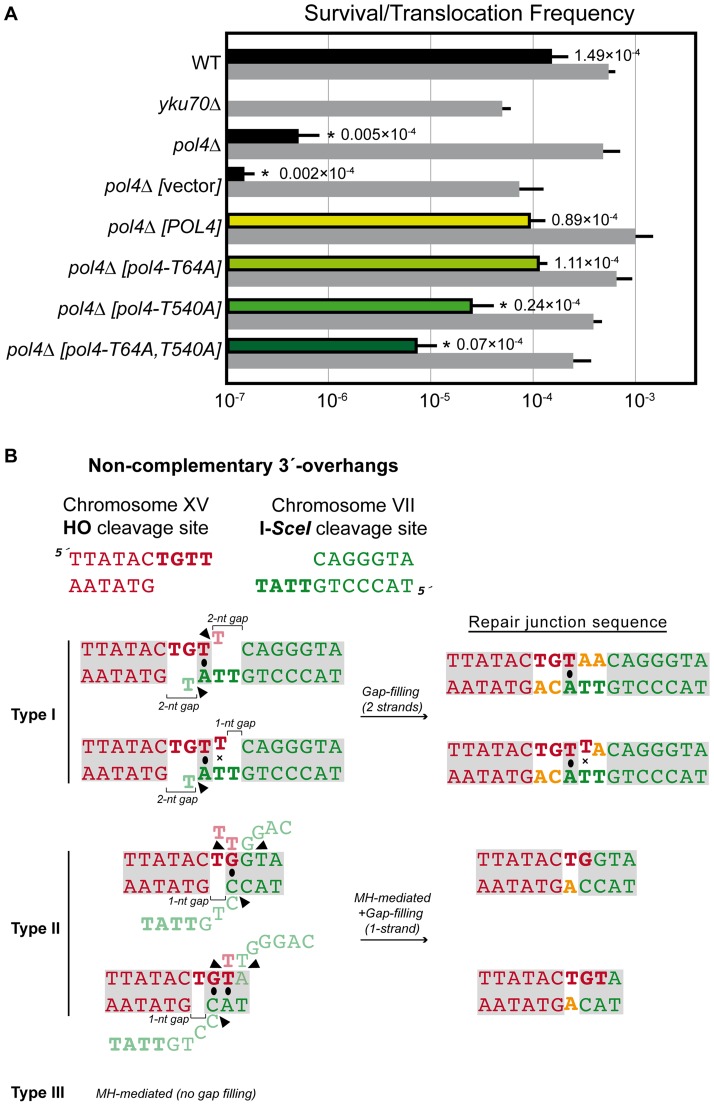
NHEJ repair types of DSBs with non-complementary ends. (A) Wild-type and indicated mutant yeast strains were subjected to two simultaneous DSBs by continuous expression of both I-*SceI* and HO by switching growth conditions from glucose- to galactose-containing media. Total cell survival (Gal/Glu, grey bars) and Leu+ translocant frequency among total cells (Gal Leu+/Glu, black, white and colored bars) are plotted on a logarithmic scale. Details as in Figure 2. (B) NHEJ repair types of DSBs with non-complementary ends. Details as in Figure 3.

Next we sought to examine the role of Pol4 in the formation of translocations in the absence of nucleotide complementarity between DNA ends to be repaired. For this purpose, we devised another system in which we introduced the I-*SceI* endonuclease cleavage site in inverse orientation with respect to the previous assay (Figure 5). Thus, concomitant DSBs produced by HO and I-*SceI* endonucleases generated 3′-protruding DNA ends that were totally non-complementary (Figure 5). In agreement with the greater difficulty of repairing such DSBs, cells carrying this new system showed lower survival frequencies compared to the previous assay (two orders of magnitude, Tables S1 and S2). Despite this, we found the same dependence on Yku70 to repair the induced DSBs (Figure 6A and Table S2). The main repair type in wild-type cells carrying this system was not mediated through partial annealing of 3′-overhanging ends and gap-filling on both strands (Type I; Table 2 and Figure 6B), as expected by the non-complementary nature of DSB ends. Instead of this, DSB repair in this new assay was favored by the use of short microhomologies around end sequences and gap-filling reactions on only one strand (Type II; Table 2 and Figure 6B). Nevertheless, the absence of Pol4 in this new assay resulted in a stronger decrease in translocation frequency with respect to wild-type (1.49 *vs.* 0.005, 300-fold decrease; Figure 6A and Table S2), as compared to the previous assay. In agreement, repair events involving gap-filling on both strands (Type I) completely disappeared in *pol4Δ*. Concomitantly, a new class of events (Type III), which were mediated by microhomology searching and did not require gap-filling, appeared in these cells (Table 2). Pol4 overexpression in *pol4*Δ cells restored translocation frequency levels (Figure 6A and Table S2) and increased type I repair events over levels found in wild-type cells (Table 2). The overexpression of Pol4 phosphomutant proteins in this new system generated the same effects observed in the previous assay. Thus, whereas *pol4*Δ [*pol4-T64A*] mutant behaved like *pol4Δ* [*POL4*] cells, both translocation frequency and repair events using 2-strand gap-filling were significantly decreased in *pol4*Δ [*pol4-T540A*] mutant cells (from 28% to 16%, p<0.005; Table 2 and Figure 6). Overall, these results indicated that the phosphorylation of Pol4-Thr^540^ by Tel1 stimulated Pol4-mediated gap-filling synthesis also during NHEJ repair of non-complementary DSBs.

**Table 2:** Repair types of DSBs with non-complementary overhangs.

Strain	Leu+ Sequenced	Type I	Type II	Type III
		*Gap-filling (2-strands)*	*MH-mediated and Gap-filling (1-strand)*	*MH-mediated no Gap-filling*
WT	25	28% (7)	72% (18)	0
*yku70Δ*	0	nd	nd	nd
*pol4Δ*	20	0	35% (7)	65% (13)
*pol4Δ* [*POL4*]	31	48% (15)	36% (11)	16% (5)
*pol4Δ* [*pol4*-*T64A*]	27	55% (15)	41% (11)	4% (1)
*pol4Δ* [*pol4*-*T540A*]	37	16%*** (6)	32% (12)	51% (19)
*pol4Δ* [*pol4*-*T64A,T540A*]	0	nd	nd	nd

Asterisks indicate statistically significant differences using a chi-squared test (*** p<0.005; *pol4Δ* [*pol4*-*T540A*] compared to *pol4Δ* [*POL4*]. nd, no data).

### DSB location has no effect on the role of Pol4-Thr^540^ phosphorylation in NHEJ

Finally, we asked whether phosphorylation of Pol4-Thr^540^ also affected DNA synthesis-mediated NHEJ of DSBs formed simultaneously in the same chromosome (*in cis*). To address this question, we used a previously described yeast assay [34], in which two I-*SceI* sites are integrated with opposing orientation on each side of the *URA3* gene in chromosome V (Figure S5). Upon continuous expression of the I-*SceI* endonuclease, almost all survivors repaired the induced DSBs by joining the two distal non-complementary DSB ends and lost the intervening *URA3* gene. This repair occurs via Pol4-mediated NHEJ [34]. Thus, we analyzed the effect of the *pol4-T540* mutant allele in the repair of these two DSBs generated *in cis* (Figure S5). As expected, DSB repair frequency decreased significantly in *pol4Δ* cells compared to wild-type (13-fold decrease, p<0.001, Figure S5). Whereas the expression of wild-type Pol4 in *pol4Δ* cells efficiently restored wild-type repair frequency, the expression of a catalytically inactive Pol4 did not (Figure S5). Of our particular interest, DSB repair frequency in *pol4-T540A* mutants decreased significantly with respect to *pol4Δ* cells expressing wild-type Pol4 (8-fold decrease, Figure S5). These results indicate that the phosphorylation of Pol4-Thr^540^ influenced gap-filling DNA synthesis during NHEJ repair independently of DSBs location.

## Discussion

In this work, we have devised yeast assays to understand the mechanisms by which DSBs generated *in vivo* in different chromosomes can be joined by NHEJ to form chromosomal translocations. These assays allow the formation of two site-specific DSBs with 3′-overhangs having either partially- or non-complementary end sequences. Breakpoint sequence analysis of translocations showed that end-joining events were mainly based on short base pairing between overhanging ends coupled to efficient Pol4-dependent gap-filling. In addition, we discovered a relevant role for Tel1 kinase in the modulation of Pol4 activity during NHEJ through the phosphorylation of Thr^540^ amino acid residue. Indeed, the phosphorylation state of this residue might have relevant structural and functional implications in the action of Pol4, promoting gap-filling DNA synthesis during NHEJ repair.

Eukaryotic cells have two different types of NHEJ, which essentially differ in their dependence on Ku proteins [7]. Our assays rely on the classical Ku-dependent NHEJ (c-NHEJ) pathway, which mainly operates on both blunt and fully complementary DSBs that can be directly ligated. Moreover, it is also able to utilize DSBs with 3′-overhanging single-stranded ends that can partially anneal. However, in these cases an additional processing of DNA ends is needed. Most of end-joining events that we recovered in our assays relied on base pairing between overhanging sequences coupled to an efficient DNA end processing. This processing frequently implied gap-filling DNA synthesis prior to ligation, and occasionally DNA end trimming. In cells carrying our systems, we also observed some NHEJ events that used short microhomologies present in sequences adjacent to DSB ends for base pairing before ligation. Nevertheless, in all these events, the extent of microhomology used for base pairing did not exceed 5-nt. Therefore, they cannot be considered as alternative (Ku-independent) NHEJ-mediated events [9]. Our assays do not permit very long DNA end resections, since an extensive degradation of intronic sequences used would impede the recovery of selectable funcional *LEU2* genes. This is in agreement with the high dependence of translocations on the presence of Yku70 that we observe.

Among the repair types analyzed in our assays, those end-joining reactions that required the filling of short gaps formed on both DNA strands showed a complete dependence on Pol4. This demonstrates the relevance of Pol4-mediated DNA synthesis in NHEJ, in agreement with previous data [28]–[32], which is a result of the special ability of Pol4 to stabilize base pairing via protein-DNA interactions when continuity of both strands is disrupted [31]. We still found NHEJ events involving gap-filling DNA synthesis on only one strand in *pol4Δ* translocants with our second system. This is probably due to the fact that base stacking interactions across broken strands can occasionally stabilize template continuity, allowing other polymerases to substitute for Pol4, as previously reported [31]–[33], [41]. The involvement of other polymerases in NHEJ when Pol4 is not present is also demonstrated by the existence of residual gap-filling repair events in *tel1*Δ *pol4*Δ double mutants in our assays. In fact, although we do not know how the lack of Tel1 could affect the action of these other polymerases during NHEJ, it is tempting to speculate that it could facilitate their activity. This would explain why the decrease of NHEJ repair generated by the absence of Pol4 is much higher in wild-type cells than in *tel1*Δ mutants. It is worth noting that Pol4 overexpression in our assays also increased the occurrence of NHEJ reactions by direct ligation. This is especially noticeable when overexpressing a dominant negative Pol4 (*pol4*Δ [*pol4*-*D367A,D369A*] mutant) and suggests that Pol4 might also act as a scaffold in some circumstances, in agreement with previous results [32]. In these cases, it could protect DNA ends from extensive resection and favor direct ligation, as has been also suggested for other polymerases [41]. Similarly, the presence of Polμ (a Pol4 orthologue) limits the resection of DNA ends at Ig genes *in vivo* during VDJ recombination in murine B cells [42].

**Figure 7:**
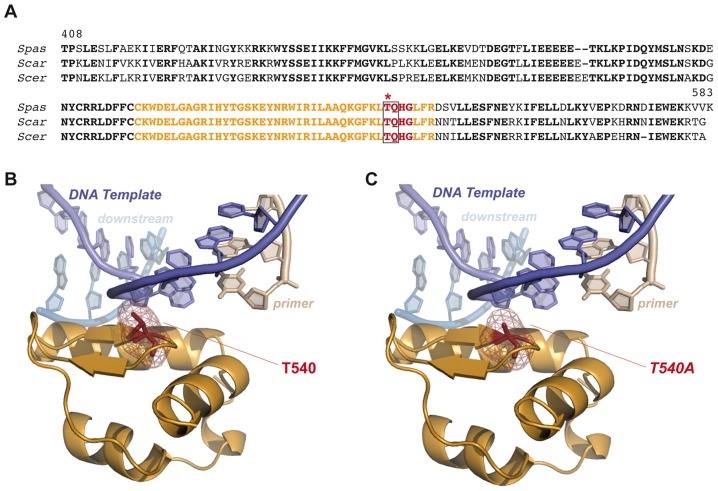
Modeling consequences of Tel1-mediated Pol4 phosphorylation at Thr^540^ amino acid residue during NHEJ. (A) Pol4 partial sequence alignment. Carboxy-terminal sequence alignment of Pol4 proteins from three different *Saccharomyces* species. Residues 540 to 543 (TQHG) are shown in red. Tel1-consensus site (TQ) is surrounded by a grey box, and Thr^540^ residue is marked with an asterisk. Amino acid residues shown in orange are specifically represented in B and C. (B) Structural modeling of yeast Pol4. Yeast Pol4 model using the crystal structure of human Polλ in a complex with a 1-nt gapped DNA and the correct incoming nucleotide (PDB:1XSN) [48]. Template, downstream and primer DNA molecules are marked. Tridimensional location of Thr^540^ is shown (in red). Only orange-coloured sequence from the amino acid alignment in (A) is shown in this tridimensional representation. (C) Modeling the effect of Thr^540^ substitution by a non-phosphorylatable alanine (*T540A*).

One of the initial events in c-NHEJ is the binding of Ku proteins to DSBs. Once Ku binds to DNA ends, they are protected from degradation and other NHEJ components can now be recruited with a high flexibility [43]. This recruitment could be directed by the complexity of DNA ends, that is, depending on their base complementarity extent. In this scenario, phosphorylation of downstream proteins emerges as a relevant mechanism to coordinate the repair process [44]. Tel1/ATM is the main kinase initially recruited to DSBs, where it phosphorylates a number of downstream effector proteins. Through the phosphorylation of some of these proteins, Tel1/ATM promotes the accurate DNA end utilization during c-NHEJ [39] and avoid formation of dangerous chromosomal rearrangements [38], [45], [46]. Our results confirm Tel1 involvement in preventing translocations and identify Pol4 as a novel target of Tel1 after DSBs generation. Interestingly, mammalian Polλ (a Pol4 orthologue) is phosphorylated by ATM in response to DNA damage [47], although the physiological significance of this phosphorylation remains to be elucidated. As shown here, Pol4 phosphorylation specifically occurs at C-terminal Thr^540^ residue. This modification may have relevant structural implications, as expected from its location in the thumb subdomain. Since Pol4 amino acid sequence is relatively well conserved (*i.e.* up to 25% amino acid identity with Polλ catalytic core), it is possible to model yeast Pol4 using the crystal structure of human Polλ forming a ternary complex with a 1-nt gapped DNA substrate and the incoming nucleotide (Figure 7) [48]. According to this model, Pol4-Thr^540^ residue would be part of a short hairpin comprising residues 540 to 543 (TQHG) that is located quite near the DNA template (Figure 7). Interestingly, an equivalent motif in human Polμ has been implicated in the correct positioning of its Loop1 structural motif and the template strand, two critical features for an efficient DNA synthesis-mediated NHEJ reaction *in vitro* (unpublished data).

From our structural model, it can be predicted that phosphorylation of Pol4-Thr^540^ by Tel1 could affect the interaction with the DNA template (Figure 7). As a consequence, this would modify the ability of Pol4 to use 3′-ended NHEJ substrates stabilized by extremely short terminal base pairing. Our data suggest that the phosphorylation of Pol4 by Tel1 may optimize Pol4 to handle DNA ends as a function of the base complementarity extent. This would enhance Pol4-mediated gap-filling activity during NHEJ repair. Supporting this hypothesis, we found that preventing Pol4 phosphorylation at Thr^540^ residue (*pol4*Δ [*pol4-T540A*] mutant) produced a significant decrease in the occurrence of translocations in our systems, mainly due to a reduced gap-filling-mediated repair of both partially- and non-complementary DSBs. Remarkably, end-bridging reactions, which involve DNA synthesis from an unpaired template to join the DSB ends, increased in the *pol4*Δ [*pol4-T540A*] mutant. This type of repair events, rare in wild-type cells, also became more visible in *tel1Δ* cells, in which Pol4-Thr^540^ residue cannot be phosphorylated. Thus, it is tempting to speculate that the increase of translocations observed in the absence of Tel1 could be, in part, a consequence of the absence of phosphorylation at Pol4-Thr^540^, which would impede an efficient gap-filling-mediated repair and favor end-bridging reactions. The combination of *tel1Δ* and *pol4Δ* mutations allowed us to get a more detailed analysis of the genetic interaction between Tel1 and Pol4. First, we observed that the overexpression of wild-type Pol4 or Pol4-*T540A* mutant in *tel1Δ pol4Δ* double mutant cells resulted in similar translocation frequency levels. This ruled out a possible negative effect of *T540A* mutation on the catalytic activity of Pol4, since Pol4-*T540A* mutant complemented *tel1Δ pol4Δ* as wild-type Pol4 did. In addition, the analysis of *pol4*Δ [*pol4-T540A*] mutants confirmed the epistasis between *tel1Δ* and *pol4-T540A* mutations, as repair types observed in double mutants were similar to those in single mutants. Again, this included a significant decrease in gap-filling-mediated repair and a concomitant increase in end-bridging repair. Finally, we also present evidence that the *pol4-T540A* mutation equally affects repair of DSBs generated both *in cis* and *in trans*. Together, our results show that phosphorylation of Pol4 by Tel1 promotes gap-filling-dependent NHEJ repair independently of the location of the DSBs. Thus, in spite of the decrease in translocations observed in the absence of Pol4 phosphorylation, we believe that such modification is, at the same time, essential to prevent NHEJ repair of DSBs in *trans*, since it also stimulates efficient gap-filling-mediated NHEJ repair of DSBs in *cis*. Indeed, in the absence of Tel1, defective DSB end tethering and resection, together with a less efficient Pol4-mediated NHEJ repair *in cis*, would lead to an increased DSB persistence and, ultimately, to an increased occurrence of chromosomal translocations.

In summary, this work uncovers a new insight during DSB repair by NHEJ, showing Pol4 to be a double-edged sword: although it primarily would contribute to repair DSBs *in cis*, it may occasionally promote the repair *in trans* generating chromosomal translocations. The finding that classical NHEJ can be another source of chromosomal rearrangements is particularly important in yeast, where it is known that simultaneous DSBs are recruited to centralized repair centers to make the repair more efficient [49]. In this process PolX polymerases could have a relevant role, as recently suggested [50]. Interestingly, the molecular features of the yeast translocations described here resemble some translocation junctions from human cancer cells, often characterized by the presence of short nucleotide deletions and/or additions as a result of NHEJ-mediated processing [51]. Therefore, this work provides further insight to the molecular mechanisms of NHEJ, and presents a new perspective to understand how chromosomal translocations are formed in cancer cells.

## Materials and Methods

### Yeast strains and plasmids

Yeast strains used in this study are listed in Table S3. All yeast strains were isogenic to W303 and contained both *HO* and I-*SCEI* genes under the *GAL1* promoter. Strains also had deleted the endogenous *LEU2* gene and *ACT1* intron. To obtain the DSB repair assay with partially-complementary ends (Figure 1) complementary oligos *Sac*II-I*Sce*I-*Sma*I-F and *Sac*II-I*Sce*I-*Sma*I-R were used (all primers used are listed in Table S4). They were annealed to generate the I-*SceI* cleavage site. This fragment was digested with *Sac*II and *Sma*I and cloned in canonical 5′-3′ orientation at the same sites of plasmid pGLB-*ACT1i*-U [52] (plasmids used are listed in Table S5). The resulting plasmid (GLB-ACT1i-U-pce) was used as a template to amplify the *GAL1p*::*leu2Δ3′*::*ACT1-iΔ3′*::I-*SceI*::*URA3* fragment by PCR. This fragment was then integrated in chromosome III of J00 strain as previously described [52]. To obtain a non-complementary ends system (Figure 5), complementary oligos *Sac*II-I*ecS*I-*Sma*I-F and *Sac*II-*IecS*I*-Sma*I-R were used along with the same strategy as described above to introduce the I-*SceI* cleavage site in a reverse orientation in plasmid pGLB-*ACT1i*-U. The corresponding *GAL1p*::*leu2Δ3′*::*ACT1-iΔ3′*::*IecS*-I::*URA3* fragment was then amplified by PCR using the oligos ADH4int-GAL1-F and ADH4int-URA3-R for its integration in chromosome VII of J00 strain. Chromosome integrations were confirmed by PCR and Southern analysis. Single- and double-deletion mutants (*pol4*Δ, y*ku70*Δ, *tel1*Δ, *tel1*Δ *pol4*Δ) were generated by PCR-based gene replacement and were confirmed by PCR and Southern analysis following standard procedures.

Full-length *POL4* DNA coding sequences were obtained by PCR amplification with primers CT-P4s and CT-P4as, which had *Cla*I and *Not*I cleavage sites, respectively. *POL4ΔBRCT* DNA sequence was obtained by PCR amplification with primers CT-P4ΔB and CT-P4as. Yeast *POL4* and *POL4ΔBRCT* overexpression plasmids were obtained by cloning the corresponding *Cla*I*-Not*I PCR fragments under the *Tet*-promoter into pCM184 plasmid. *POL4* single (*T64A*, *T540A*) and double (*T64A,T540A* and *D367A,D369A*) mutations in pCM184 plasmid were obtained by site-directed mutagenesis using the corresponding mutated primers. All mutated overexpression plasmids were verified by DNA sequencing. Wild-type and point mutant *T540A* versions were also tagged with Flag epitope by PCR amplification using primers CT-P4s and p4FLAGnot-as, together with the corresponding pCM184-[*POL4*] plasmids as a template. The different PCR products were digested with *Cla*I and *Not*I and then cloned into pCM184. Wild-type and mutant (*T64A*, *T540A*, *T64A-T540A*) *POL4* versions were also fused to 6×His-tag epitope by subcloning the corresponding *BamH*I-*Not*I fragments from pCM184 into pET28c(+) vector (Novagen).

### Determination of recombination frequencies

Determination of recombination frequencies was performed as described previously [52] with some modifications. Briefly, at least four independent colonies were grown until reaching the logarithmic phase in glucose-containing synthetic complete medium (SC) and then switched to glycerol-lactate (SC-3% glycerol/2% lactate). Cells in glycerol-lactate were allowed to complete one cell cycle. In such conditions, they naturally accumulate in the G1 phase of the cell cycle, allowing the DSB induction to take place when NHEJ is predominant. Appropriate dilutions were then plated on SC (glu) to determine the total cell number before DSB induction by the addition of 2% galactose to liquid cultures. After galactose addition, yeast cultures were incubated for 4 h in order to quickly induce the DSBs in G1-accumulated cells. After this incubation time, appropriate dilutions were plated onto complete galactose-containing media with (SGal) or without (SGal-Leu) leucine. Cell survival was determined by dividing the number of colonies growing on SGal after DSB repair by the number of colonies growing on SC before DSB induction. The frequency of translocations was determined by dividing the number of colonies growing on SGal-Leu by the number of colonies growing on SC (total cells). This parameter was used as a reference value to compare different strains.

To determine recombination frequencies in the repair of DSBs generated *in cis* we used a previously reported yeast genetic assay [34]. Briefly, appropriate dilutions of cells from overnight cultures in glycerol-lactate without uracil were spread on glucose- and galactose-containing plates. Survivor colonies on galactose-containing plates were replica-plated on SC plates containing 5-FOA (USBiological), to discriminate between Ura^−^ and Ura^+^ cells. The frequency of DSB repair involving the loss of the *URA3* gene was determined by dividing the number of colonies growing on SC+5-FOA by the number of colonies growing on SC.

Statistical significance of translocation frequencies in mutant strains was evaluated with the Mann-Whitney test compared to wild-type cells (in mutant strains y*ku70Δ*, *pol4Δ*, *tel1Δ* and *tel1*Δ *pol4*Δ), or compared to *pol4Δ* [*POL4*] cells (in *pol4Δ* cells overexpressing mutant Pol4 versions). The distribution of repair events obtained in the different mutant strains was compared to that of wild-type strain using the Chi-square test. The distribution of repair events obtained in *pol4Δ* cells overexpressing mutant Pol4 versions was compared to that of *pol4* [*POL4*] strain using the same test.

### Immunoprecipitation, immunoblotting and kinase assays

Cells were grown up to the exponential phase and were then synchronized at G1 by addition of α-factor. DSBs were induced by addition of 100 µg/ml Zeocin (Invitrogen). After 1 h incubation, cells were broken using glass-beads in lysis buffer (20 mM Hepes-KOH pH 7.5, 150 mM NaCl, 10% glycerol, 0.1% Tween-20, 1 mM phenylmethylculphonyl fluoride, Complete protease inhibitor cocktail (Roche), PhosSTOP phosphatase inhibitor cocktail (Roche)) for 20 min at 4°C. Extracts were clarified twice by centrifugation. Flag-Pol4 proteins were immunoprecipitated from supernatants with anti-Flag M2 antibody (Sigma) coupled to Protein G Sepharose 4 Fast Flow (GE Healthcare) in lysis buffer overnight at 4°C on a rotating wheel. Sepharose-bound proteins were centrifugated, washed extensively with lysis buffer and eluted in Laemmli buffer. Anti-Flag M2 antibody (Sigma) and anti-phospho [S/T]Q ATM/ATR Substrate Antibody (Cell Signaling) were used in immunoblotting experiments following standard procedures. For *in vitro* kinase assays, we partially purified recombinant His-tagged Pol4 proteins using Ni-NTA agarose (Qiagen) following manufacturer's instructions. Tel1-HA was immunoprecipitated from cells previously transformed with plasmid pKR5, which encodes an HA-tagged *TEL1* gene [40]. Control non-transformed cells were assayed in parallel to obtain HA-immunoprecipitates without HA-Tel1 enrichment that were used as a negative control in kinase assays. Both transformed and non-transformed cells were grown to exponential phase and broken using glass-beads in lysis buffer (25 mM MOPS pH 7.2, 15 mM EGTA, 0.1% NP-40, 150 mM KCl, 1 mM DTT, protease inhibitor cocktail (Sigma), 1 mM phenylmethylsulfonyl fluoride). Extracts were clarified by centrifugation and HA-tagged Tel1 was immunoprecipitated from soluble fractions with anti-HA antibodies (Roche). Immunocomplexes were collected with Protein G-coupled DynaBeads (Life Technologies) and used in kinase assays as described previously [40].

### Amino acid sequence comparisons and 3D-modelling

Multiple alignment of the three *Saccharomyces* Pol4 DNA polymerases was done using MULTALIN (http://multalin.toulouse.inra.fr/multalin). Pol4 amino acid sequence was modeled using human Polλ PDB coordinates and Swiss-Model software (http://swissmodel.expasy.org). For tridimensional structure extrapolations, we compared this Pol4 model with crystal structure of human Polλ in a ternary complex with a 1-nt gapped DNA substrate and the incoming nucleotide (PDB code:1XSN) [48]. This was obtained from the Protein Data Bank (http://www.rcsb.org/pdb). Pol4-Thr^540^ residue and the corresponding point mutation was identified by using PyMol software (http://pymol.org/).

### Miscellaneous

Chromosomal breakpoint analysis by PCR and DNA sequencing, and molecular karyotyping of Leu+ translocants by pulsed-field gel electrophoresis were performed as previously described [35], [52]. Breakpoint sequences from all sequenced Leu+ translocants are shown in Figure S2.

## Supporting Information

Figure S1 Molecular karyotype of wild-type Leu+ translocants. (*Upper*) Scheme of the assay. (*Lower*) PFGE analysis of 12 independent wild-type translocants. Parental strain (P) is shown as a reference. Gels were stained with ethidium bromide (*left*) and analyzed by Southern using an *HYG* specific probe (*right*). Electrophoretic mobility of natural yeast chromosomes is indicated on the left. After DSBs induction and Leu+ selection, two new translocated chromosomes can be detected (*t*III/XV and *t*XV/III, marked with red triangles). Parental chromosomes III and XV are marked in bold. In all samples analyzed the *HYG* signal disappeared from parental chromosome XV and was specifically detected in the larger translocated chromosome (*t*XV/III). Chromosomes XV and VII have the same electrophoretic mobility in the experimental conditions used here.
(TIF)

Figure S2 Breakpoint sequences from wild-type and mutant Leu+ translocants. Only canonical 5′-to-3′ upper strand is shown. The 4-nucleotide long 3′-protruding single-stranded DNA ends generated after both I-*SceI* and HO cleavage are shown in bold. Inserted nucleotides are represented in red. Nucleotides in blue boxes indicate sequence microhomologies. Nucleotides processed by mismatch repair are shown in blue. *n* indicates the number of independent clones of each strain sequenced.
(PDF)

Figure S3 Leu+ translocation frequencies in *pol4Δ* cells expressing *POL4* alleles from the endogenous *POL4* promoter. Indicated yeast strains were subjected to two simultaneous DSBs by continuous expression of both I-*SceI* and HO by switching growth conditions from glucose- to galactose-containing media. Cell survival (Gal/Glu, grey bars) and Leu+ translocant frequency among total cells (Gal Leu+/Glu, black, white and colored bars) are plotted on a logarithmic scale. Data represent the median plus standard deviation from at least four independent experiments. Statistically significant lower values with respect to *pol4Δ* [e*POL4*] complemented strains are marked with an asterisk (*p<0.001 by the Mann-Whitney test).
(TIF)

Figure S4 Partial purification of Pol4 polymerase and Tel1 kinase. (A) Purification of Pol4 proteins. His-tagged Pol4 proteins were partially purified using Ni-NTA agarose, separated in 8% SDS-PAGE and Coomassie stained. A 70-kDa main product corresponding to the expected electrophoretic mobility of Pol4 proteins is indicated. A smaller contaminant protein, marked with an asterisk, was co-purified in all samples. (B) Immunoprecipitation of Tel1 from yeast. HA-tagged yeast Tel1 kinase was immunoprecipitated with anti-HA antibodies from cells transformed with a plasmid encoding *TEL1::HA*, as previously described [40]. Immunoprecipitated HA-Tel1 was immunodetected by Western using anti-HA antibodies and is indicated with an arrow.
(TIF)

Figure S5 Yeast assay to analyze NHEJ-mediated repair of DSBs in *cis*. (A) Scheme of the assay. Two I-*SceI* sites are integrated with opposing orientation on each side of the *URA3* gene in the chromosome V. After endonuclease induction, two permanent non-complementary DSBs are produced. NHEJ-mediated repair of distal non-complementary DSB ends generates the loss of the intervening *URA3* gene [34]. (B) Effect of Pol4 mutants in NHEJ-mediated repair of DSBs *in cis*. Wild-type and indicated mutants were subjected to two simultaneous DSBs *in cis* by continuous expression of I-*SceI* by switching growth conditions from glucose- to galactose-containing media. *POL4* alleles were expressed from *POL4* endogenous promoter. DSB repair frequency is plotted on a logarithmic scale. Data represent the median plus standard deviation obtained from four independent experiments. Values significantly lower than either wild-type (WT) or *pol4Δ* [*POL4*] strains are indicated (p<0.001 by the Mann-Whitney test).
(TIF)

Table S1 Survival and translocation frequencies after repair of DSBs with partially-complementary overhangs.
(PDF)

Table S2 Survival and translocation frequencies after repair of DSBs with non-complementary overhangs.
(PDF)

Table S3 Yeast strains used in this study.
(PDF)

Table S4 Primers used in this study.
(PDF)

Table S5 Plasmids used in this study.
(PDF)
