# Supplementary figures and images for: Yeast Pol4 Promotes Tel1-Regulated Chromosomal Translocations
Source: PLoS Genet. 2013 Jul 18;9(7):e1003656. doi: 10.1371/journal.pgen.1003656 (PMC3715435; doi:10.1371/journal.pgen.1003656)

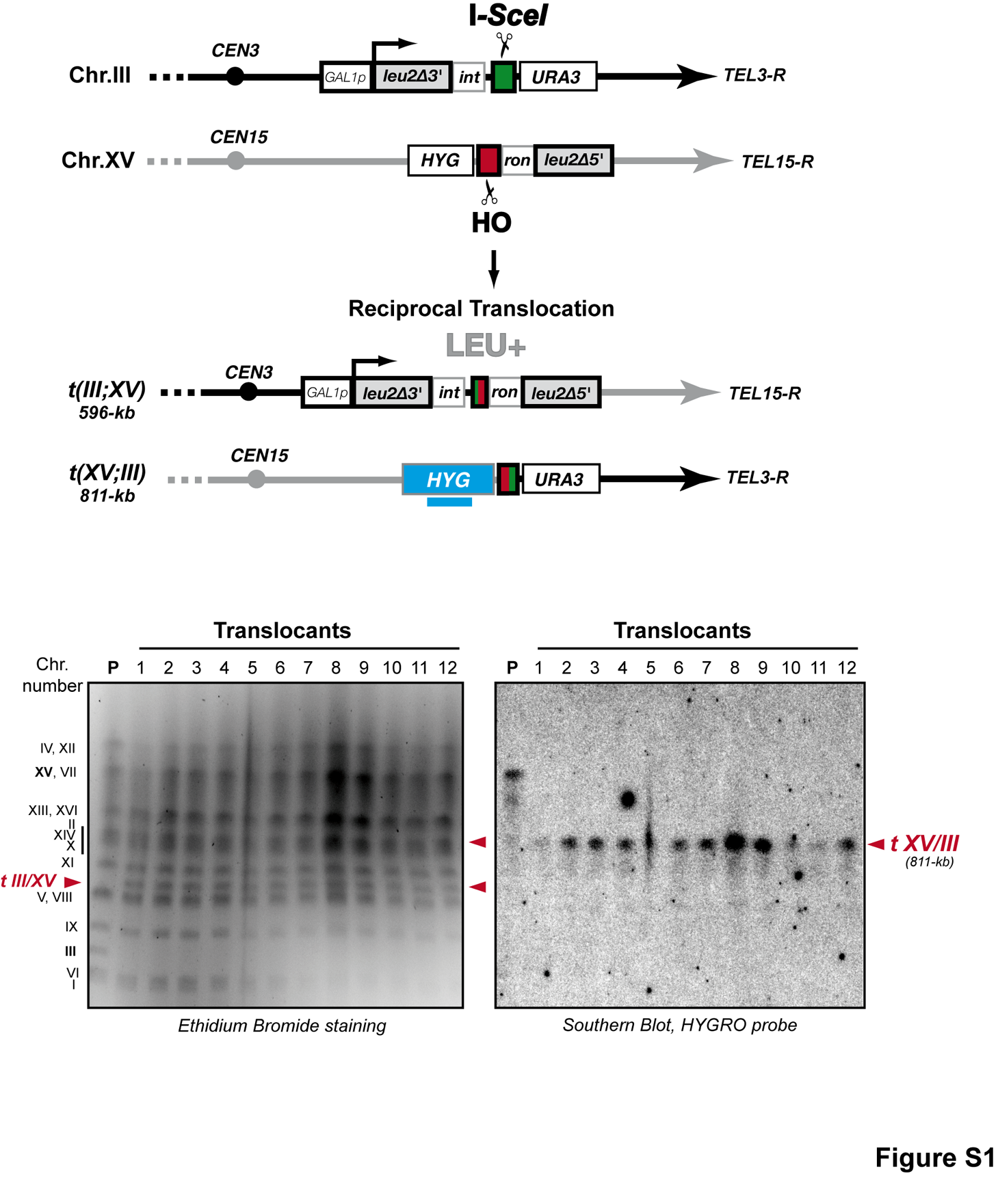

Supplement: Figure S1 — Molecular karyotype of wild-type Leu+ translocants. (Upper) Scheme of the assay. (Lower) PFGE analysis of 12 independent wild-type translocants. Parental strain (P) is shown as a reference. Gels were stained with ethidium bromide (left) and analyzed by Southern using an HYG specific probe (right). Electrophoretic mobility of natural yeast chromosomes is indicated on the left. After DSBs induction and Leu+ selection, two new translocated chromosomes can be detected (tIII/XV and tXV/III, marked with red triangles). Parental chromosomes III and XV are marked in bold. In all samples analyzed the HYG signal disappeared from parental chromosome XV and was specifically detected in the larger translocated chromosome (tXV/III). Chromosomes XV and VII have the same electrophoretic mobility in the experimental conditions used here. (TIF) [file pgen.1003656.s001.tif]

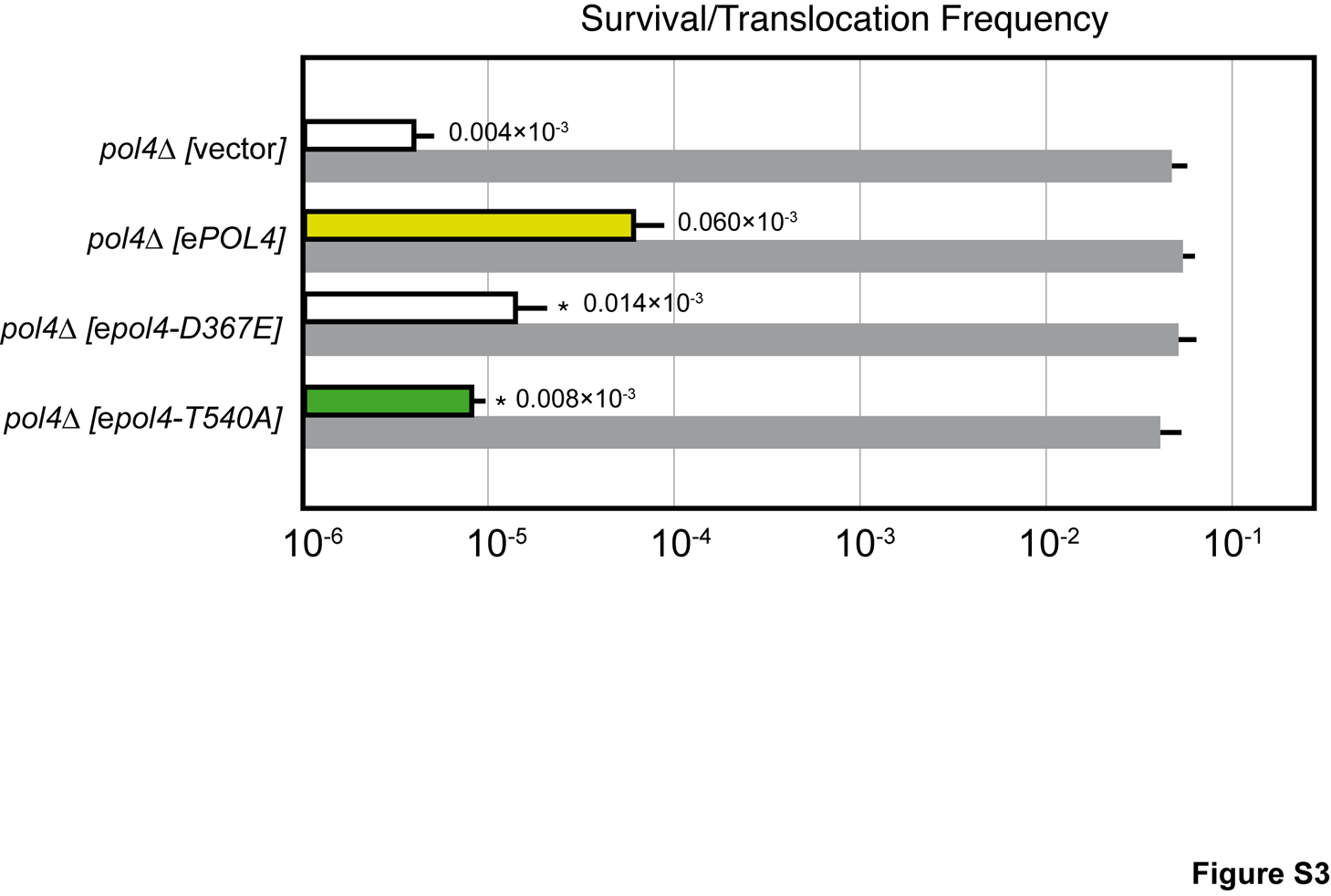

Supplement: Figure S3 — Leu+ translocation frequencies in pol4Δ cells expressing POL4 alleles from the endogenous POL4 promoter. Indicated yeast strains were subjected to two simultaneous DSBs by continuous expression of both I-SceI and HO by switching growth conditions from glucose- to galactose-containing media. Cell survival (Gal/Glu, grey bars) and Leu+ translocant frequency among total cells (Gal Leu+/Glu, black, white and colored bars) are plotted on a logarithmic scale. Data represent the median plus standard deviation from at least four independent experiments. Statistically significant lower values with respect to pol4Δ [ePOL4] complemented strains are marked with an asterisk (*p<0.001 by the Mann-Whitney test). (TIF) [file pgen.1003656.s003.tif]

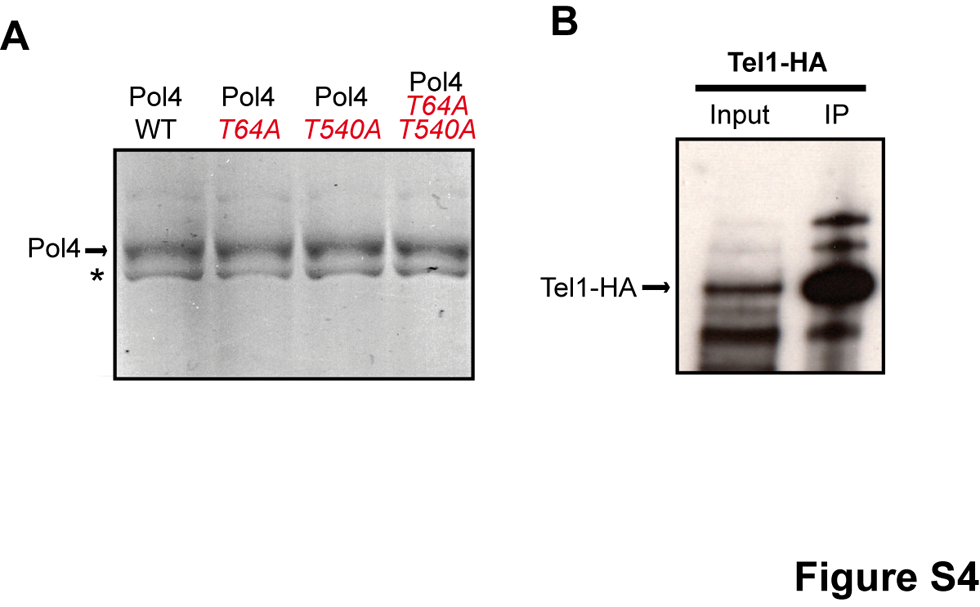

Supplement: Figure S4 — Partial purification of Pol4 polymerase and Tel1 kinase. (A) Purification of Pol4 proteins. His-tagged Pol4 proteins were partially purified using Ni-NTA agarose, separated in 8% SDS-PAGE and Coomassie stained. A 70-kDa main product corresponding to the expected electrophoretic mobility of Pol4 proteins is indicated. A smaller contaminant protein, marked with an asterisk, was co-purified in all samples. (B) Immunoprecipitation of Tel1 from yeast. HA-tagged yeast Tel1 kinase was immunoprecipitated with anti-HA antibodies from cells transformed with a plasmid encoding TEL1::HA, as previously described [40]. Immunoprecipitated HA-Tel1 was immunodetected by Western using anti-HA antibodies and is indicated with an arrow. (TIF) [file pgen.1003656.s004.tif]

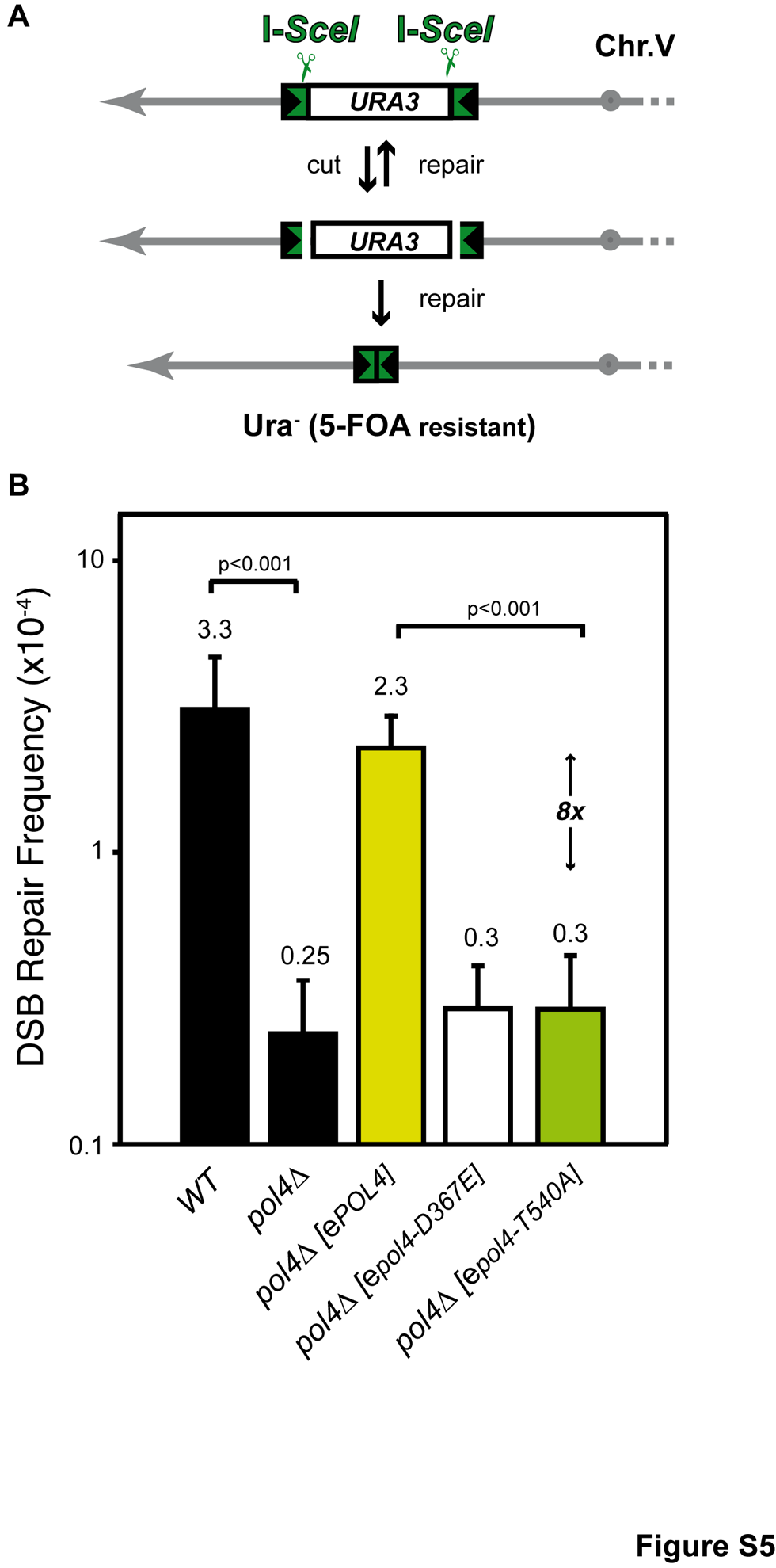

Supplement: Figure S5 — Yeast assay to analyze NHEJ-mediated repair of DSBs in cis. (A) Scheme of the assay. Two I-SceI sites are integrated with opposing orientation on each side of the URA3 gene in the chromosome V. After endonuclease induction, two permanent non-complementary DSBs are produced. NHEJ-mediated repair of distal non-complementary DSB ends generates the loss of the intervening URA3 gene [34]. (B) Effect of Pol4 mutants in NHEJ-mediated repair of DSBs in cis. Wild-type and indicated mutants were subjected to two simultaneous DSBs in cis by continuous expression of I-SceI by switching growth conditions from glucose- to galactose-containing media. POL4 alleles were expressed from POL4 endogenous promoter. DSB repair frequency is plotted on a logarithmic scale. Data represent the median plus standard deviation obtained from four independent experiments. Values significantly lower than either wild-type (WT) or pol4Δ [POL4] strains are indicated (p<0.001 by the Mann-Whitney test). (TIF) [file pgen.1003656.s005.tif]
