# Supplementary material for: Yeast Pol4 Promotes Tel1-Regulated Chromosomal Translocations
Source: PLoS Genet. 2013 Jul 18;9(7):e1003656. doi: 10.1371/journal.pgen.1003656 (PMC3715435; doi:10.1371/journal.pgen.1003656)
Supplement: Figure S2 — Breakpoint sequences from wild-type and mutant Leu+ translocants. Only canonical 5′-to-3′ upper strand is shown. The 4-nucleotide long 3′-protruding single-stranded DNA ends generated after both I-SceI and HO cleavage are shown in bold. Inserted nucleotides are represented in red. Nucleotides in blue boxes indicate sequence microhomologies. Nucleotides processed by mismatch repair are shown in blue. n indicates the number of independent clones of each strain sequenced. (PDF) [file pgen.1003656.s002.pdf]

Partially-complementary 3´-overhangs

I *SceI* cleavage site (Chromosome III)

5´GGATGTAGGG**ATAA**
CCTACATCCC

HO cleavage site (Chromosome XV)

GTATAATTTTA
TTGTCATATTAAAAT5´

wild type *(n=24)*

w9	GGATGTAGGG**ATAA**	CAGTATAATTTTA
w10	GGATGTAGGG**ATAA**	CAGTATAATTTTA
w11	GGATGTAGGG**ATAA**	CAGTATAATTTTA
w12	GGATGTAGGG**ATAA**	CAGTATAATTTTA
w13	GGATGTAGGG**ATAA**	CAGTATAATTTTA
w16	GGATGTAGGG**ATAA**	CAGTATAATTTTA
w17	GGATGTAGGG**ATAA**	CAGTATAATTTTA
w20	GGATGTAGGG**ATAA**	CAGTATAATTTTA
w5	GGATGTAGGG**ATAA**	CAGTATAATTTTA
w7	GGATGTAGGG**ATAA**	CAGTATAATTTTA
w8	GGATGTAGGG**ATAA**	CAGTATAATTTTA
wS2	GGATGTAGGG**ATAA**	CAGTATAATTTTA
wS4	GGATGTAGGG**ATAA**	CAGTATAATTTTA
w1	GGATGTAGGG**ATA**	CAGTATAATTTTA
w4	GGATGTAGGG**ATA**	CAGTATAATTTTA
w18	GGATGTAGGG**ATA**	CAGTATAATTTTA
w2	GGATGTAGGG**ATAA**	TTTTA
w3	GGATGTAGGG**ATAA**	TTTTA
w6	GGATGTAGGG**ATAA**	TTTTA
w14	GGATGTAGGG**ATAA**	TTTTA
w19	GGATGTAGGG**ATAA**	TTTTA
w15	GGATGTAGGG**ATA**	GTATAATTTTA
wS5	GGATGTAGGG**ACA**	GTATAATTTTA
wS3	GGATGTAGG	AACAGTATAATTTTA

***pol4*Δ POL4** *(n=35)*

P4+1	GGATGTAGGG**ATAA**	CAGTATAATTTTA
P4+3	GGATGTAGGG**ATAA**	CAGTATAATTTTA
P4+5	GGATGTAGGG**ATAA**	CAGTATAATTTTA
P4+6	GGATGTAGGG**ATAA**	CAGTATAATTTTA
P4+8	GGATGTAGGG**ATAA**	CAGTATAATTTTA
P4+9	GGATGTAGGG**ATAA**	CAGTATAATTTTA
P4+12	GGATGTAGGG**ATAA**	CAGTATAATTTTA
P4+2	GGATGTAGGG**ATAA**	CAGTATAATTTTA
PS+17	GGATGTAGGG**ATAA**	CAGTATAATTTTA
PS+18	GGATGTAGGG**ATAA**	CAGTATAATTTTA
PS+20	GGATGTAGGG**ATAA**	CAGTATAATTTTA
PS+22	GGATGTAGGG**ATAA**	CAGTATAATTTTA
PS+23	GGATGTAGGG**ATAA**	CAGTATAATTTTA
JAP4+1	GGATGTAGGG**ATAA**	CAGTATAATTTTA
JAP4+2	GGATGTAGGG**ATAA**	CAGTATAATTTTA
JAP4+5	GGATGTAGGG**ATAA**	CAGTATAATTTTA
JAP4+11	GGATGTAGGG**ATAA**	CAGTATAATTTTA
P4+7	GGATGTAGGG**ATA**	CAGTATAATTTTA
P4+6	GGATGTAGGG**ATA**	CAGTATAATTTTA
P4+4	GGATGTAGGG**ATA**	CAGTATAATTTTA
JAP4+4	GGATGTAGGG**ATA**	CAGTATAATTTTA
JAP4+12	GGATGTAGGG**ATA**	CAGTATAATTTTA
JAP4+13	GGATGTAGGG**ATA**	CAGTATAATTTTA
JAP4+14	GGATGTAGGG**ATA**	CAGTATAATTTTA
JAP4+9	GGATGTAGGG**ATA**	GTATAATTTTA
JAP4+10	GGATGTAGGG**ATA**	GTATAATTTTA
P4+2	GGATGTAGGG**ATA**	GTATAATTTTA
P4+4	GGATGTAGGG**ATA**	GTATAATTTTA
P4+5	GGATGTAGGG**ATA**	GTATAATTTTA
P4+3	GGATGTAGGG**ACA**	GTATAATTTTA
P4+11	GGATGTAGGG**ACA**	GTATAATTTTA
PS+21	GGATGTAGGG**ACA**	GTATAATTTTA
P4+1	GGATGTAGGG**AACA**	GTATAATTTTA
PS+19	GGATGTAGGG**A**	GTATAATTTTA
P4+10	GGATGTAGGG**ATAA**	TTTTA

***pol4*Δ** *(n=21)*

p41	GGATGTAGGG**ATAA**	TTTTA
p42	GGATGTAGGG**ATAA**	TTTTA
p45	GGATGTAGGG**ATAA**	TTTTA
p46	GGATGTAGGG**ATAA**	TTTTA
p47	GGATGTAGGG**ATAA**	TTTTA
p49	GGATGTAGGG**ATAA**	TTTTA
p50	GGATGTAGGG**ATAA**	TTTTA
p4-6	GGATGTAGGG**ATAA**	TTTTA
pS11	GGATGTAGGG**ATAA**	TTTTA
pS15	GGATGTAGGG**ATAA**	TTTTA
pS16	GGATGTAGGG**ATAA**	TTTTA
wS12	GGATGTAGGG**AT**	(48 nt del)
p43	GGATGTAGGG**ATA**	GTATAATTTTA
p44	GGATGTAGGG**ATA**	GTATAATTTTA
p50	GGATGTAGGG**ATA**	GTATAATTTTA
p4-4	GGATGTAGGG**ATA**	GTATAATTTTA
p4-5	GGATGTAGGG**ATA**	GTATAATTTTA
pS10	GGATGTAGGG**ATA**	GTATAATTTTA
pS14	GGATGTAGGG**ATA**	GTATAATTTTA
p4-1	GGATGTAGGG**ACA**	GTATAATTTTA
wS9	GGATGTAGG	AACAGTATAATTTTA

***pol4*Δ *POL4 BRCT*Δ** *(n=18)*

B6	GGATGTAGGG**ATAA**	TTTTA
B5	GGATGTAGGG**ATAA**	TTTTA
B9	GGATGTAGGG**ATAA**	TTTTA
B12b	GGATGTAGGG**ATAA**	TTTTA
B14	GGATGTAGGG**ATAA**	TTTTA
B14b	GGATGTAGGG**ATAA**	TTTTA
B7	GGATGTAGGG**ATA**	GTATAATTTTA
B8	GGATGTAGGG**ATA**	GTATAATTTTA
B10	GGATGTAGGG**ATA**	GTATAATTTTA
B10b	GGATGTAGGG**ATA**	GTATAATTTTA
B11	GGATGTAGGG**ATA**	GTATAATTTTA
B11b	GGATGTAGGG**ATA**	GTATAATTTTA
B13	GGATGTAGGG**ATA**	GTATAATTTTA
Bs11	GGATGTAGGG**ATA**	GTATAATTTTA
Bs13	GGATGTAGGG**ATA**	GTATAATTTTA
Bs12	GGATGTAGG	AGTATAATTTTA
B12	GGATGTAGG	AACAGTATAATTTTA
Bs14		GTATAATTTTA

Partially-complementary 3´-overhangs

I-*SceI* cleavage site (Chromosome III)

5´GGATGTAGGGATAA
CCTACATCCC

HO cleavage site (Chromosome XV)

GTATAATTTTA
TTGTCATATTAAAAT 5´

*pol4*Δ [POL4-*T64A*] (n=35)

64-1	GGATGTAGGGATAA	CAGTATAATTTTA
64-2	GGATGTAGGGATAA	CAGTATAATTTTA
64-4	GGATGTAGGGATAA	CAGTATAATTTTA
64-8	GGATGTAGGGATAA	CAGTATAATTTTA
64-9	GGATGTAGGGATAA	CAGTATAATTTTA
64-10	GGATGTAGGGATAA	CAGTATAATTTTA
64-11	GGATGTAGGGATAA	CAGTATAATTTTA
64-12	GGATGTAGGGATAA	CAGTATAATTTTA
64S33	GGATGTAGGGATAA	CAGTATAATTTTA
64S34	GGATGTAGGGATAA	CAGTATAATTTTA
64S35	GGATGTAGGGATAA	CAGTATAATTTTA
64S36	GGATGTAGGGATAA	CAGTATAATTTTA
64S37	GGATGTAGGGATAA	CAGTATAATTTTA
64S39	GGATGTAGGGATAA	CAGTATAATTTTA
64S40	GGATGTAGGGATAA	CAGTATAATTTTA
64S15	GGATGTAGGGATAA	CAGTATAATTTTA
64S16	GGATGTAGGGATAA	CAGTATAATTTTA
64S17	GGATGTAGGGATAA	CAGTATAATTTTA
T64A2	GGATGTAGGGATAA	CAGTATAATTTTA
T64A7	GGATGTAGGGATAA	CAGTATAATTTTA
T64A12	GGATGTAGGGATAA	CAGTATAATTTTA
T64A13	GGATGTAGGGAAA	CAGTATAATTTTA
T64A10	GGATGTAGGGATA	CAGTATAATTTTA
64-5	GGATGTAGGGATA	CAGTATAATTTTA
T64A1	GGATGTAGGGATA	CAGTATAATTTTA
T64A3	GGATGTAGGGATA	CAGTATAATTTTA
T64A9	GGATGTAGGG	CAGTATAATTTTA
64-7	GGATGTAGGGATA	GTATAATTTTA
T64A6	GGATGTAGGGATA	GTATAATTTTA
T64A5	GGATGTAGGGACA	GTATAATTTTA
64S38	GGATGTAGGGAACA	GTATAATTTTA
T64A4	GGATGTAGGGATAA	GTATAATTTTA
T64A8	GGATGTAGG	AACAGTATAATTTTA
T64A11	GGATGTAGG	AACAGTATAATTTTA
64-6		AACAGTATAATTTTA

*pol4*Δ [POL4-*T540A*] (n=36)

540-4	GGATGTAGGGATAA	CAGTATAATTTTA
540-5	GGATGTAGGGATAA	CAGTATAATTTTA
540-6	GGATGTAGGGATAA	CAGTATAATTTTA
540-8	GGATGTAGGGATAA	CAGTATAATTTTA
540-11	GGATGTAGGGATAA	CAGTATAATTTTA
540-14	GGATGTAGGGATAA	CAGTATAATTTTA
540-15	GGATGTAGGGATAA	CAGTATAATTTTA
T540A4	GGATGTAGGGATAA	CAGTATAATTTTA
T540A5	GGATGTAGGGATAA	CAGTATAATTTTA
T540A7	GGATGTAGGGATAA	CAGTATAATTTTA
T540A11	GGATGTAGGGATAA	CAGTATAATTTTA
T540A9	GGATGTAGGGATA	CAGTATAATTTTA
540S29	GGATGTA	CAGTATAATTTTA
540-9	GGATGTAGGGATAA	TTTTA
540S26	GGATGTAGGGATAA	TTTTA
540S27	GGATGTAGGGATAA	TTTTA
540S28	GGATGTAGGGATAA	TTTTA
540S30	GGATGTAGGGATAA	TTTTA
540S25	GGATGTAG	TATAATTTTA
T540A1	GGATGTAGGGATA	GTATAATTTTA
T540A3	GGATGTAGGGATA	GTATAATTTTA
T540A6	GGATGTAGGGATA	GTATAATTTTA
T540A8	GGATGTAGGGATA	GTATAATTTTA
T540A10	GGATGTAGGGATA	GTATAATTTTA
T540A12	GGATGTAGGGATA	GTATAATTTTA
T540A13	GGATGTAGGGATA	GTATAATTTTA
540-10	GGATGTAGGGATA	GTATAATTTTA
540S32	GGATGTAGGGACA	GTATAATTTTA
540-13	GGATGTAGGGATAA	GTATAATTTTA
540-1	GGATGTAGG	AACAGTATAATTTTA
540-2	GGATGTAGG	AACAGTATAATTTTA
540-3	GGATGTAGG	AACAGTATAATTTTA
540-7	GGATGTAGG	AACAGTATAATTTTA
540-12	GGATGTAGG	AACAGTATAATTTTA
540S31	GGATGTAGG	AACAGTATAATTTTA
T540A2	GGATGTAGG	AACAGTATAATTTTA

*pol4*Δ [POL4-*D367A,D369A*] (n=17)

DxS42	GGATGTAGGGATA	GTATAATTTTA
DxS44	GGATGTAGGGATA	GTATAATTTTA
DxS46	GGATGTAGGGATA	GTATAATTTTA
DxS47	GGATGTAGGGATA	GTATAATTTTA
DxS48	GGATGTAGGGATA	GTATAATTTTA
DxS22	GGATGTAGGGATA	GTATAATTTTA
DxS23	GGATGTAGGGATA	GTATAATTTTA
Dx1	GGATGTAGGGATA	GTATAATTTTA
Dx3	GGATGTAGGGATA	GTATAATTTTA
Dx6	GGATGTAGGGATA	GTATAATTTTA
Dx2	GGATGTAGGGACA	GTATAATTTTA
Dx4	GGATGTAGGGACA	GTATAATTTTA
DxS21	GGATGTAGGGATAA	TTTTA
DxS43	GGATGTAGGG	TATAATTTTA
DxS-19	GGATGTA	TAATTTTA
DxS41	GGATGTAGG	AACAGTATAATTTTA
DxS20	GGATGTAGG	AACAGTATAATTTTA

*tel1*Δ (n=27)

tel1	GGATGTAGGGATAA	CAGTATAATTTTA
tel2	GGATGTAGGGATAA	CAGTATAATTTTA
tel14	GGATGTAGGGATAA	CAGTATAATTTTA
tel4*	GGATGTAGGGATAA	CAGTATAATTTTA
tel5*	GGATGTAGGGATAA	CAGTATAATTTTA
telS4	GGATGTAGGGATA	CAGTATAATTTTA
telS5	GGATGTAGGGATA	CAGTATAATTTTA
tel3*	GGATGTA	CAGTATAATTTTA
tel2*		CAGTATAATTTTA
telS1	GGATGTA	GTATAATTTTA
tel6	GGATGTAGGGATAA	TTTTA
tel13	GGATGTAGGGATAA	TTTTA
tel9	GGATGTAGGGATAA	TTTTA
telS6	GGATGTAGGGATAA	TTTTA
tel4	GGATGTAGGGATA	TAATTTTA
tel10	GGATGTAGGGATA	GTATAATTTTA
tel11	GGATGTAGGGATA	GTATAATTTTA
tel8	GGATGTAGGGACA	GTATAATTTTA
telS3	GGATGTAGGGACA	GTATAATTTTA
tel3	GGATGTAGG	AACAGTATAATTTTA
tel12	GGATGTAGG	AACAGTATAATTTTA
telS2	GGATGTAGG	AACAGTATAATTTTA
tel6*	GGATGTAGG	AACAGTATAATTTTA
tel15	G	CAGTATAATTTTA
tel5	GGATGTAGGGA	AGTATAATTTTA
tel16	GGATGTAGGGA	AGTATAATTTTA
tel7	GGATGTA	AGTATAATTTTA

Partially-complementary 3´-overhangs

```
I-SceI cleavage site           HO cleavage site
(Chromosome III)               (Chromosome XV)
5´GGATGTAGGGATAA                    GTATAATTTTA
  CCTACATCCC                    TTGTCATATTAAAAT5´
```

*tel1*Δ *pol4*Δ *(n=23)*

```
#11 GGATGTAGGGATAA      CAGTATAATTTTA
#15 GGATGTAGGGATAA      CAGTATAATTTTA
#19 GGATGTAGGGATAA      CAGTATAATTTTA
#2  GGATGTAGGGATA       CAGTATAATTTTA
#6  GGATGTAGGGATA       CAGTATAATTTTA
#7  GGA                 CAGTATAATTTTA
#18 GGATGTAGGGATAA               TTTTA
#12 GGATGTAGGGATAA               TTTTA
#20 GGATGTAGGGATAA               TTTTA
#17 GGATGTAG                 TATAATTTTA
#1  GGATGTAGGGATA           GTATAATTTTA
#9  GGATGTAGGGATA           GTATAATTTTA
#21 GGATGTAGGGATA           GTATAATTTTA
#22 GGATGTAGGGACA           GTATAATTTTA
#23 GGATGTAGGGACA           GTATAATTTTA
#5  GGATGTAGGGACA           GTATAATTTTA
#10 GGATGTAGGGACA           GTATAATTTTA
#13 GGATGTAGGGACA           GTATAATTTTA
#8  GGATGTAGGGA             GTATAATTTTA
#16 GGATGTAGGGA             GTATAATTTTA
#3  GGATGTAGG           AACAGTATAATTTTA
#4  GGATGTAGG           AACAGTATAATTTTA
#14 GGATGTAGG           AACAGTATAATTTTA
```

*tel1*Δ *pol4*Δ [POL4] *(n=30)*

```
#1  GGATGTAGGGATAA      CAGTATAATTTTA
#3  GGATGTAGGGATAA      CAGTATAATTTTA
#7  GGATGTAGGGATAA      CAGTATAATTTTA
#13 GGATGTAGGGATAA      CAGTATAATTTTA
#14 GGATGTAGGGATAA      CAGTATAATTTTA
#16 GGATGTAGGGATAA      CAGTATAATTTTA
#17 GGATGTAGGGATAA      CAGTATAATTTTA
#21 GGATGTAGGGATAA      CAGTATAATTTTA
#22 GGATGTAGGGATAA      CAGTATAATTTTA
#26 GGATGTAGGGATAA      CAGTATAATTTTA
#2  GGATGTAGGGATA       CAGTATAATTTTA
#15 GGATGTAGGGATA       CAGTATAATTTTA
#20 GGATGTAGGGATA       CAGTATAATTTTA
#19 GGATGTAGGGATAA               TTTTA
#4  GGATGTAGGGATAA               TTTTA
#9  GGATGTAGGG               TATAATTTTA
#10 GGATGTAGGG               TATAATTTTA
#8  GGATGTAG                 TATAATTTTA
#18 GGATGTA                    TAATTTTA
#6  GGA–GTA                    TAATTTTA
#12 GGATGTAGGGATA           GTATAATTTTA
#23 GGATGTAGGGACA           GTATAATTTTA
#5  GGATGTAGGGACA           GTATAATTTTA
#29 GGATGTAGGGACA           GTATAATTTTA
#30 GGATGTAGGGACA           GTATAATTTTA
#31 GGATGTAGGGACA           GTATAATTTTA
#32 GGATGTAGGGACA           GTATAATTTTA
#11 GGATGTAGG           AACAGTATAATTTTA
#27 GGATGTAGG           AACAGTATAATTTTA
#28 GGATGTAGG           AACAGTATAATTTTA
```

*tel1*Δ *pol4*Δ [*pol4-T540A*] *(n=30)*

```
#1  GGATGTAGGGATAA      CAGTATAATTTTA
#14 GGATGTAGGGATAA      CAGTATAATTTTA
#18 GGATGTAGGGATAA      CAGTATAATTTTA
#23 GGATGTAGGGATAA      CAGTATAATTTTA
#25 GGATGTAGGGATAA      CAGTATAATTTTA
#17 GGATGTAGGGATA       CAGTATAATTTTA
#22 GGATGTAGGGATA       CAGTATAATTTTA
#24 GGATGTAGGGATA       CAGTATAATTTTA
#5  GGATGTAGGGATAA               TTTTA
#7  GGATGTAGGGATAA               TTTTA
#8  GGATGTAGGGATAA               TTTTA
#9  GGATGTAGGGATAA               TTTTA
#12 GGATGTAGGGATAA               TTTTA
#20 GGATGTAGGGATAA               TTTTA
#3  GGATGTAGGGATA           GTATAATTTTA
#4  GGATGTAGGGATA           GTATAATTTTA
#13 GGATGTAGGGATA           GTATAATTTTA
#15 GGATGTAGGGATA           GTATAATTTTA
#19 GGATGTAGGGATA           GTATAATTTTA
#29 GGATGTAGGGATA           GTATAATTTTA
#26 GGATGTAGGGACA           GTATAATTTTA
#30 GGATGTAGGGACA           GTATAATTTTA
#31 GGATGTAGGGACA           GTATAATTTTA
#32 GGATGTAGGGACA           GTATAATTTTA
#27 GGATGTAGGGA             GTATAATTTTA
#2  GGATGTAGG           AACAGTATAATTTTA
#11 GGATGTAGG           AACAGTATAATTTTA
#16 GGATGTAGG           AACAGTATAATTTTA
#10 GGATGTA                AGTATAATTTTA
#21                        AGTATAATTTTA
```

Non-complementary 3´-overhangs

HO cleavage site (Chromosome XV) — I *SceI* cleavage site (Chromosome VII)

```
GGGTTTATAAAATTATACTGTT          CAGGGTAATA
CCCAAATATTTTAATATG          TATTGTCCCATTAT
```

wild type *(n=25)*

```
w9    GGGTTTATAAAATTATACTGT       AACAGGGTAATA
w16   GGGTTTATAAAATTATACTGT       AACAGGGTAATA
w12   GGGTTTATAAAATTATACTGT       AACAGGGTAATA
w15   GGGTTTATAAAATTATACTGT       AACAGGGTAATA
S49   GGGTTTATAAAATTATACT         AACAGGGTAATA
w20   GGGTTTATAAAATTATACTGT       TACAGGGTAATA
S50   GGGTTTATAAAATTATACTGT       TACAGGGTAATA
w10   GGGTTTATAAAATTATACTGTT              AATA
w13   GGGTTTATAAAATTATACTGTT              AATA
w4    GGGTTTATAAAATTATACTGT               AATA
w6    GGGTTTATAAAATTATACTGT               AATA
w7    GGGTTTATAAAATTATACTGT               AATA
w12   GGGTTTATAAAATTATACTGT               AATA
w17   GGGTTTATAAAATTATACTGT               AATA
w18   GGGTTTATAAAATTATACTGT               AATA
S52   GGGTTTATAAAATTATACTGT               AATA
S53   GGGTTTATAAAATTATACTGT               AATA
w1    GGGTTTATAAAATTATACTG             GGTAATA
w2    GGGTTTATAAAATTATACTG              GTAATA
w5    GGGTTTATAAAATTATACTG              GTAATA
w3    GGGTTTATAAAATTATACTG              GTAATA
w8    GGGTTTATAAAATTATACTG              GTAATA
w11   GGGTTTATAAAATTATACTG              GTAATA
w19   GGGTTTATAAAATTATACTG              GTAATA
S51   GGGTTTATAAAATTATACTG              GTAATA
```

***pol4*Δ** *(n=20)*

```
p49    GGGTTTATAAAATTATACTG            GGTAATA
p45    GGGTTTATAAAATTATACTG             GTAATA
p46    GGGTTTATAAAATTATACTG             GTAATA
p44    GGGTTTATAAAATTATACTGT              AATA
p50    GGGTTTATAAAATTATACTGT              AATA
Bp4-3  GGGTTTATAAAATTATAC           CAGGGTAATA
p47    GGGTTTATAAAATTATA           ACAGGGTAATA
pS25   GGGTTTATAAAATTATA           ACAGGGTAATA
p41    GGGTTTATAA                   CAGGGTAATA
p42    GGG                              GTAATA
pS24   GGG                              GTAATA
Bp4-2  GGG                              GTAATA
p43    GGG                               TAATA
p48    GGG                               TAATA
pS18   GGG                               TAATA
pS26   GGG                               TAATA
Bp4-1  GGG                               TAATA
Bp4-4  GGG                               TAATA
Bp4-5  GGG                               TAATA
Bp4-6  GG
```

***pol4*Δ POL4** *(n=31)*

```
P4+8'    GGGTTTATAAAATTATACTGTT      AACAGGGTAATA
BP4+10   GGGTTTATAAAATTATACTGTT      AACAGGGTAATA
P4+4     GGGTTTATAAAATTATACTGT     ATAACAGGGTAATA
BP4+12   GGGTTTATAAAATTATACTGT       AACAGGGTAATA
P4+1     GGGTTTATAAAATTATACTGT       AACAGGGTAATA
P4+2     GGGTTTATAAAATTATACTGT       AACAGGGTAATA
P4+12    GGGTTTATAAAATTATACTGT       AACAGGGTAATA
P4+11    GGGTTTATAAAATTATACTGT       AACAGGGTAATA
S63      GGGTTTATAAAATTATACTGT       AACAGGGTAATA
S64      GGGTTTATAAAATTATACTGT       AACAGGGTAATA
S62      GGGTTTATAAAATTATACTGT       AACAGGGTAATA
P4+13    GGGTTTATAAAATTATACTGT       TACAGGGTAATA
P4+10    GGGTTTATAAAATTATACTG        AACAGGGTAATA
S61      GGGTTTATAAAATTATA            ACAGGGTAATA
P4+1'    GGGTTTATAAAATTATA            ACAGGGTAATA
P4+3'    GGGTTTATAAAATTATACTGT          AGGGTAATA
P4+4'    GGGTTTATAAAATTATACTGT            GGTAATA
P4+5'    GGGTTTATAAAATTATACTG          CAGGGTAATA
S60      GGGTTTATAAAATTATACTGT               AATA
BP4+9    GGGTTTATAAAATTATACTGT               AATA
BP4+11   GGGTTTATAAAATTATACTGT               AATA
S59      GGGTTTATAAAATTATACTG             GGTAATA
P4+3     GGGTTTATAAAATTATACTG             GGTAATA
P4+2'    GGGTTTATAAAATTATACTG             GGTAATA
P4+6     GGGTTTATAAAATTATACTG              GTAATA
P4+7'    GGGTTTATAAAATTATACTG              GTAATA
P4+6'    GGGTTTATAAAA                  CAGGGTAATA
P4+7     GGGTTTATAAAATTATAC              GGGTAATA
P4+8     GGGTTTATAAAATTATA               GGGTAATA
S58      GGGTTTATAAAATTATA               GGGTAATA
S57      GGG                                TAATA
```

Non-complementary 3′-overhangs

HO cleavage site (Chromosome XV)	I *SceI* cleavage site (Chromosome VII)
GGGTTTATAAAATTATAC**TGTT**	CAGGGTAATA
CCCAAATATTTTAATATG	**TATT**GTCCCATTAT

*pol4*Δ [POL4-*T64A*] *(n=27)*

S64-10	GGGTTTATAAAATTATAC**TGTT**	**AA**CAGGGTAATA
S64-11	GGGTTTATAAAATTATAC**TGTT**	**AA**CAGGGTAATA
S64-6	GGGTTTATAAAATTATAC**TGTT**	**AA**CAGGGTAATA
S64-8	GGGTTTATAAAATTATAC**TGT**	**ATAA**CAGGGTAATA
64A8	GGGTTTATAAAATTATAC**TGT**	**ATAA**CAGGGTAATA
S64-1	GGGTTTATAAAATTATAC**TGT**	**AA**CAGGGTAATA
S64-2	GGGTTTATAAAATTATAC**TGT**	**AA**CAGGGTAATA
S64-3	GGGTTTATAAAATTATAC**TGT**	**AA**CAGGGTAATA
S64-7	GGGTTTATAAAATTATAC**TGT**	**AA**CAGGGTAATA
S64-14	GGGTTTATAAAATTATAC**TGT**	**AA**CAGGGTAATA
S64-15	GGGTTTATAAAATTATAC**TGT**	**AA**CAGGGTAATA
64A5	GGGTTTATAAAATTATAC**TGT**	**AA**CAGGGTAATA
64A9	GGGTTTATAAAATTATAC**TGT**	**AA**CAGGGTAATA
64A12	GGGTTTATAAAATTATAC**TGT**	**TA**CAGGGTAATA
64A1	GGGTTTATAAAATTATAC**TGT**	**TA**CAGGGTAATA
S64-5	GGGTTTATAAAATTAT	**AA**CAGGGTAATA
S64-4	GGGTTTATAAAATTATAC**TGT**	A
64A10	GGGTTTATAAAATTATAC**TG**	GGTAATA
64A11	GGGTTTATAAAATTATAC**TG**	GTAATA
64A3	GGGTTTATAAAATTATAC**TG**	GTAATA
S64-12	GGGTTTATAAAATTATAC**TGT**	AATA
S64-13	GGGTTTATAAAATTATAC**TGT**	AATA
64A2	GGGTTTATAAAATTATAC**TGT**	AATA
64A6	GGGTTTATAAAATTATAC**TGT**	AATA
64A7	GGGTTTATAAAATTATAC**TGT**	AATA
64A1	GGGTTTATAAAATTATAC**TGT**	AATA
S64-9	GGGTTTATAAAATTATAC**TGT**	GTAATA

*pol4*Δ [POL4-*T540A*] *(n=37)*

S65	GGGTTTATAAAATTATAC**TGT**	**AA**CAGGGTAATA
S67	GGGTTTATAAAATTATAC**TGT**	**AA**CAGGGTAATA
540A6	GGGTTTATAAAATTATAC**TGT**	**AA**CAGGGTAATA
540A10	GGGTTTATAAAATTATAC**TGT**	**AA**CAGGGTAATA
S69	GGGTTTATAAAATTATAC**TGT**	**AA**CAGGGTAATA
S540-3	GGGTTTATAAAATTAT	**AA**CAGGGTAATA
540A11	GGGTTTATAAAATTATAC**TG**	CAGGGTAATA
540A4	GGGTTTATAAAATTATAC**TGT**	GGGTAATA
S72	GGGTTTATAAAATTATAC**TGTT**	AATA
S540-2	GGGTTTATAAAATTATAC**TGTT**	AATA
S66	GGGTTTATAAAATTATAC**TGT**	AATA
540A3	GGGTTTATAAAATTATAC**TGT**	AATA
540A7	GGGTTTATAAAATTATAC**TGT**	AATA
540A12	GGGTTTATAAAATTATAC**TGT**	AATA
540-8	GGGTTTATAAAATTATAC**TG**	GTAATA
S68	GGGTTTATAAAATTATAC**TG**	GGTAATA
540A1	GGGTTTATAAAATTATAC**TG**	GGTAATA
S540-1	GGGTTTATAAAATTATAC**T**	**A**CAGGGTAATA
S70	GGGTTTATAAAATTATA	CAGGGTAATA
540-2	GGGTTTATAAAATTATA	A
540-101	GGGTTTATAAAATTATA	A
540A2	GGGTTTATAA	CAGGGTAATA
540A5	GGGTTTATAA	CAGGGTAATA
540A8	GGGTTTATAA	CAGGGTAATA
pS27	GGGTTTATAA	CAGGGTAATA
540A9	GGGTTTATAA	CAGGGTAATA
540-6	GGGTTTATAAAA	CAGGGTAATA
540-51	GGGT	GTAATA
540-102	GGG	
540-1	GGG	TAATA
540-3	GGG	TAATA
540-4	GGG	TAATA
540-7	GGG	TAATA
540-52	GGG	TAATA
540-91	GGG	TAATA
540-92	GGG	TAATA
540-93	GGG	TAATA
