# Supplementary material for: Yeast Pol4 Promotes Tel1-Regulated Chromosomal Translocations
Source: PLoS Genet. 2013 Jul 18;9(7):e1003656. doi: 10.1371/journal.pgen.1003656 (PMC3715435; doi:10.1371/journal.pgen.1003656)
Supplement: Table S1 — Survival and translocation frequencies after repair of DSBs with partially-complementary overhangs. (PDF) [file pgen.1003656.s006.pdf]

Table S1. Survival and repair frequencies of DSBs with partially-complementary overhangs

**Strain**	**Survival frequency (Gal/Glu) $\times 10^{-1}$ ± s.d.**	**Leu+ over Total cells (Glu) $\times 10^{-3}$ ± s.d.**
WT	0.51 ± 0.07	0.27 ± 0.12
WT [vector]	nd	0.04 ± 0.01
yku70Δ	0.37 ± 0.17	nd
pol4Δ	0.42 ± 0.05	0.010 ± 0.003
pol4Δ [vector]	0.49 ± 0.10	0.004 ± 0.001
pol4Δ [*POL4*]	0.78 ± 0.28	0.40 ± 0.11
pol4Δ [*pol4-D367A,D369A*]	0.30 ± 0.07	0.18 ± 0.05
pol4Δ [*pol4ΔBRCT*]	0.75 ± 0.22	0.004 ± 0.001
pol4Δ [*pol4-T64A*]	0.57 ± 0.14	0.35 ± 0.09
pol4Δ [*pol4-T540A*]	0.70 ± 0.17	0.13 ± 0.02
pol4Δ [*pol4-T64A,T540A*]	0.70 ± 0.09	0.11 ± 0.01
tel1Δ	0.47 ± 0.14	2.99 ± 0.50
tel1Δ [vector]	0.54 ± 0.15	1.29 ± 0.48
tel1Δ pol4Δ	0.42 ± 0.10	0.71 ± 0.18
tel1Δ pol4Δ [vector]	0.75 ± 0.10	0.49 ± 0.07
tel1Δ pol4Δ [*POL4*]	0.68 ± 0.05	0.88 ± 0.44
tel1Δ pol4Δ [*pol4-T540A*]	0.72 ± 0.21	0.82 ± 0.44
