# Supplementary material for: Yeast Pol4 Promotes Tel1-Regulated Chromosomal Translocations
Source: PLoS Genet. 2013 Jul 18;9(7):e1003656. doi: 10.1371/journal.pgen.1003656 (PMC3715435; doi:10.1371/journal.pgen.1003656)
Supplement: Table S2 — Survival and translocation frequencies after repair of DSBs with non-complementary overhangs. (PDF) [file pgen.1003656.s007.pdf]

Table S2. Survival and repair frequencies of DSBs with non-complementary overhangs

**Strain**	**Survival frequency (Gal/Glu) (×10^{-3}) ± s.d.**	**Leu+ over Total cells (Glu) ×10^{-4} ± s.d.**
WT	0.55 ± 0.06	1.49 ± 0.55
WT [vector]	nd	0.12 ± 0.05
yku70Δ	0.30 ± 0.08	nd
pol4Δ	0.45 ± 0.25	0.005 ± 0.003
pol4Δ [vector]	0.10 ± 0.05	0.002 ± 0.0003
pol4Δ [*POL4*]	0.95 ± 0.37	0.89 ± 0.35
pol4Δ [*pol4-T64A*]	0.61 ± 0.27	1.11 ± 0.22
pol4Δ [*pol4-T540A*]	0.37 ± 0.09	0.24 ± 0.15
pol4Δ [*pol4-T64A,T540A*]	0.23 ± 0.11	0.07 ± 0.04
