# Supplementary material for: Yeast Pol4 Promotes Tel1-Regulated Chromosomal Translocations
Source: PLoS Genet. 2013 Jul 18;9(7):e1003656. doi: 10.1371/journal.pgen.1003656 (PMC3715435; doi:10.1371/journal.pgen.1003656)
Supplement: Table S3 — Yeast strains used in this study. (PDF) [file pgen.1003656.s008.pdf]

Table S3. Yeast strains used in this study

Strain	Genotype	Reference
J00	*MAT**a-inc** ade2-1 can1-100 his3-11,15 leu2Δ::SFA1 trp1-1 ura3-1 ade3::GAL-HO lys2Δ::GAL1p-ISCEI ACT1Δi HYG-HO::ACT1-iΔ5´::Leu2Δ5´*	Ruiz *et al.* 2009
J4A-1	*MAT**a-inc** ade2-1 can1-100 his3-11,15 leu2Δ::SFA1 trp1-1 ura3-1 ade3::GAL-HO lys2Δ::GAL1p-ISCEI ACT1Δi ChrIII-GAL1p::leu2Δ3´::ACT1-iΔ3´::ISceI::URA3 ChrXV-HYG::HO::ACT1-iΔ5´::leu2Δ5´*	This study
J4A-2	*MAT**a-inc** ade2-1 can1-100 his3-11,15 leu2Δ::SFA1 trp1-1 ura3-1 ade3::GAL-HO lys2Δ::GAL1p-ISCEI ACT1Δi ChrIII-GAL1p::leu2Δ3´::ACT1-iΔ3´::ISceI::URA3 ChrXV-HYG::HO::ACT1-iΔ5´::leu2Δ5´ pol4Δ::natMX4*	This study
J4A-3	*MAT**a-inc** ade2-1 can1-100 his3-11,15 leu2Δ::SFA1 trp1-1 ura3-1 ade3::GAL-HO lys2Δ::GAL1p-ISCEI ACT1Δi ChrIII-GAL1p::leu2Δ3´::ACT1-iΔ3´::ISceI::URA3 ChrXV-HYG::HO::ACT1-iΔ5´::leu2Δ5´ ku70Δ::kanMX4*	This study
J4A-4	*MAT**a-inc** ade2-1 can1-100 his3-11,15 leu2Δ::SFA1 trp1-1 ura3-1 ade3::GAL-HO lys2Δ::GAL1p-ISCEI ACT1Δi ChrIII-GAL1p::leu2Δ3´::ACT1-iΔ3´::ISceI::URA3 ChrXV-HYG::HO::ACT1-iΔ5´::leu2Δ5´ tel1Δ::kanMX4*	This study
J4A-5	*MAT**a-inc** ade2-1 can1-100 his3-11,15 leu2Δ::SFA1 trp1-1 ura3-1 ade3::GAL-HO lys2Δ::GAL1p-ISCEI ACT1Δi ChrIII-GAL1p::leu2Δ3´::ACT1-iΔ3´::ISceI::URA3 ChrXV-HYG::HO::ACT1-iΔ5´::leu2Δ5´ pol4Δ::natMX4 tel1Δ::kanMX4*	This study
B6C-1	*MAT**a-inc** ade2-1 can1-100 his3-11,15 leu2Δ::SFA1 trp1-1 ura3-1 ade3::GAL-HO lys2Δ::GAL1p-ISCEI ACT1Δi ChrVII-GAL1p::leu2Δ3´::ACT1-iΔ3´::ISceI::URA3 ChrXV-HYG::HO::ACT1-iΔ5´::leu2Δ5´*	This study
B6C-2	*MAT**a-inc** ade2-1 can1-100 his3-11,15 leu2Δ::SFA1 trp1-1 ura3-1 ade3::GAL-HO lys2Δ::GAL1p-ISCEI ACT1Δi ChrVII-GAL1p::leu2Δ3´::ACT1-iΔ3´::ISceI::URA3 ChrXV-HYG::HO::ACT1-iΔ5´::leu2Δ5´ pol4Δ::natMX4*	This study
B6C-3	*MAT**a-inc** ade2-1 can1-100 his3-11,15 leu2Δ::SFA1 trp1-1 ura3-1 ade3::GAL-HO lys2Δ::GAL1p-ISCEI ACT1Δi ChrVII-GAL1p::leu2Δ3´::ACT1-iΔ3´::ISceI::URA3 ChrXV-HYG::HO::ACT1-iΔ5´::leu2Δ5´ ku70Δ::kanMX4*	This study
Lev516	*MAT**a** bar1-Δ lys2::pGAL-ISCEI ISceI::URA3::ISceI*	Pardo and Marcand, 2008
Lev517	*MAT**a** bar1-Δ lys2::pGAL-ISCEI ISceI::URA3::ISceI pol4Δ::natMX4*	Pardo and Marcand, 2008
