# Supplementary material for: Yeast Pol4 Promotes Tel1-Regulated Chromosomal Translocations
Source: PLoS Genet. 2013 Jul 18;9(7):e1003656. doi: 10.1371/journal.pgen.1003656 (PMC3715435; doi:10.1371/journal.pgen.1003656)
Supplement: Table S4 — Primers used in this study. (PDF) [file pgen.1003656.s009.pdf]

Table S4.	Primers used in this study
**Primers**	**Sequence (5´-3´)**
CT-P4s	CTATGAatcgatATGTCTCTAAAGGGTAAAT
CT-P4as	TTGATCgcggccgcTTATGCAGTTTTTTTTTCCCATTCG
CT-P4ΔB	CTATGAatcgatATGAAAGATGATTTG
T64A-s	ATGCATTTGgCTCAGAAAGAT
T64A-as	ATCTTTCTGAGcCAAATGCAT
T540A-s	TTCAAGCTTgCACAACACGG
T540A-as	CCGTGTTGTGcAAGCTTGAA
D367A,D369A-s	TGTGGTGcCATTGcTCTTTTATTTTTC
D367A,D369A-as	GAAAAATAAAAGAgCAATGgCACCACA
p4FLAGnot-as	TTGATCgcggccgcTTAGATGTTGTCATCGTCATCTTTATAATCTAATGCAGTTTTTTTTTCC
ADH4int-GAL1-F	TTGGTTTGCCACCTGCTTTGACTGCTGCTACTGGTCTAGAAAGTACGGATTAGAAGCCGC
ADH4int-URA3-R	GGCCTTCTTGTCTTTACCGTCTTTGTATGCAGCGACTAAGAGCTTTTCAATTCAATTCAT
Sac2-ISce1-Sma1-F	GGATTCCGCGGAGTGAGGATGTAGGGATAACAGGGTAATATACCACCACCCGGGTAGC
Sac2-ISce1-Sma1-R	GCTACCCGGGTGGTGGTATATTACCCTGTTATCCCTACATCCTCACTCCGCGGAATCCGC
Sac2-1ecSI-Sma1-F	GGATTCCGCGGTGGTGGTATATTACCCTGTTATCCCTACATCCTCACTCCCGGGTAGC
Sac2-1ecSI-Sma1-R	GCTACCCGGGAGTGAGGATGTAGGGATAACAGGGTAATATACCACCACCGCGGAATCCGC
