# Supplementary material for: Yeast Pol4 Promotes Tel1-Regulated Chromosomal Translocations
Source: PLoS Genet. 2013 Jul 18;9(7):e1003656. doi: 10.1371/journal.pgen.1003656 (PMC3715435; doi:10.1371/journal.pgen.1003656)
Supplement: Table S5 — Plasmids used in this study. (PDF) [file pgen.1003656.s010.pdf]

Table S5. **Plasmids used in this study**

**Plamid**	**Relevant features**	**Reference**
GLB-*ACT1*i-U	*pRS314 plasmid carrying the fragment GAL1p::leu2Δ3´::ACT1-iΔ3´::URA3*	Ruiz *et al.* 2009
GLB-ACT1i-U-pce	*pRS314 plasmid carrying the fragment GAL1p::leu2Δ3´::ACT1-iΔ3´::I-SceI::URA3*	This study
GLB-ACT1i-U-nce	*pRS314 plasmid carrying the fragment GAL1p::leu2Δ3´::ACT1-iΔ3´::IecSI::URA3*	This study
pCM-*POL4*	*Full-lenght Pol4 cloned at pCM184 ClaI and NotI sites*	This study
pCM-*POL4*(*D367A,D369A*)	*Full-lenght Pol4 with the double mutation D367A and D369A cloned at pCM184 ClaI and NotI sites*	This study
pCM-*POL4ΔB*	*Pol4 lacking the BRCT domain, cloned at pCM184 ClaI and NotI sites*	This study
pCM-*POL4*(*T64A*)	*Full-lenght Pol4 with the point mutation T64A, cloned at pCM184 ClaI and NotI sites*	This study
pCM-*POL4*(*T540A*)	*Full-lenght Pol4 with the point mutation T540A, cloned at pCM184 ClaI and NotI sites*	This study
pCM-*POL4*(*T64A,T540A*)	*Full-lenght Pol4 with the double mutation T64A and T540A, cloned at pCM184 ClaI and NotI sites*	This study
pCM-*POL4*#F	*Full-lenght Pol4 with one copy of the FLAG epitope fused to the C-terminus, cloned at pCM184 ClaI and NotI sites*	This study
pCM-*POL4*(*T540A*)#F	*Full-lenght Pol4 with one copy of the FLAG epitope fused to the C-terminus and the mutation T540A, cloned at pCM184 ClaI and NotI sites*	This study
pET-*POL4*	*Full-lenght Pol4 cloned in BamHI and NotI of pET28c(+) plasmid. Subcloned from pCM-POL4*	This study
pET-*POL4*(*T64A*)	*Full-lenght Pol4 with the point mutation T64A, cloned in BamHI and NotI of pET28c(+) plasmid. Subcloned from pCM-POL4*	This study
pET-*POL4*(*T540A*)	*Full-lenght Pol4 with the point mutation T540A, cloned in BamHI and NotI of pET28c(+) plasmid. Subcloned from pCM-POL4*	This study
pET-*POL4*(*T64A,T540A*)	*Full-lenght Pol4 with the double mutation T64A and T540A, cloned in BamHI and NotI of pET28c(+) plasmid. Subcloned from pCM-POL4*	This study
pRS314-*POL4*	*Full-lenght Pol4 cloned at pRS314 yeast expression plasmid under its physiological promoter*	Pardo and Marcand, 2006
pRS314-*POL4* (*D367E*)	*Full-lenght Pol4, with the point mutation D367E, cloned at pRS314 yeast expression plasmid under its physiological promoter*	Pardo and Marcand, 2006
pRS314-*POL4*(*T540A*)	*Full-lenght Pol4, with the point mutation T540A, cloned at pRS314 yeast expression plasmid under its physiological promoter*	This study
